# Chemical Architecture and Applications of Nucleic Acid Derivatives Containing 1,2,3-Triazole Functionalities Synthesized via Click Chemistry

**DOI:** 10.3390/molecules171112665

**Published:** 2012-10-26

**Authors:** Tim Efthymiou, Wei Gong, Jean-Paul Desaulniers

**Affiliations:** Faculty of Science, University of Ontario Institute of Technology, 2000 Simcoe St N, Oshawa, ON L1H 7K4, Canada

**Keywords:** click chemistry, triazole, CuAAC, nucleic acids, oligonucleotides, bioconjugation, copper-catalyzed, Huisgen dipolar cycloaddition, azide, alkyne

## Abstract

There is considerable attention directed at chemically modifying nucleic acids with robust functional groups in order to alter their properties. Since the breakthrough of copper-assisted azide-alkyne cycloadditions (CuAAC), there have been several reports describing the synthesis and properties of novel triazole-modified nucleic acid derivatives for potential downstream DNA- and RNA-based applications. This review will focus on highlighting representative novel nucleic acid molecular structures that have been synthesized via the “click” azide-alkyne cycloaddition. Many of these derivatives show compatibility for various applications that involve enzymatic transformation, nucleic acid hybridization, molecular tagging and purification, and gene silencing. The details of these applications are discussed. In conclusion, the future of nucleic acid analogues functionalized with triazoles is promising.

## 1. Introduction

Since its discovery by Meldal and Sharpless in 2001 [1], the Cu(I)-catalyzed adaptation of Huisgen’s 1,3-dipolar [3 + 2] azide-alkyne (CuAAC) cycloaddition has not only earned the title of “click” reaction, but has nearly become synonomous with the term due its speed, versatility, simplicity and its broad applications [2,3,4,5]. This particular click cycloaddition has had a considerable impact on chemical transformations leading to the production of new materials [6,7] and libraries of molecules with applications in biological systems [3]. The CuAAC reaction is an ideal bioorthogonal transformation because it can be performed under various environmental conditions, is high-yielding and expedient, the azide and alkyne moieties are physiologically inert [8], and the regio- and stereospecificity of the triazole product are tightly controlled [1].

In order to form ideal candidates for pharmaceuticals and novel biological research tools, nucleic acids and their oligomers often benefit from chemical modification. For oligonucleotides, chemical modification can be used to mitigate certain problematic features inherent in their native structure such as the polyanionic backbone and their susceptibility to nuclease-mediated cleavage [9]. In addition, chemical modification can be used to append novel functionalities on the nucleic acid molecule for other downstream biological applications. Such applications include, but are not limited to the field of molecular diagnostics which encompasses the synthesis of DNA microarrays [10,11,12], molecular probes [13,14], antisense oligonucleotides (ASOs) [15,16], and short-interfering RNAs (siRNAs) [17,18,19]. In addition, nucleic acid derivatives are constantly being designed, synthesized and modified for use as antiviral and anti-tumor therapies, and for the regulation of gene expression. Antiviral nucleoside analogues (ANAs), ASOs and siRNAs have been researched as potential therapeutic agents to overcome viral infections [20,21,22], or the expression of problematic genes in diseased cells [23]. There are examples of ANAs and ASOs which are commercially available [24,25], whereas the pursuit of siRNAs exhibiting properties which present them as viable pharmaceutical candidates is still underway. Some other examples of emerging biologically active nucleic acid derivatives include synthetic ribozymes [26], unique aptamers [27,28], and triplex forming oligonucleotides (TFOs) [29].

Due to their structure, there are three main areas within nucleic acids which are commonly altered through chemical modification: the ribose sugar, the nitrogenous base, and the phosphodiester internucleotide linkage. Although there are many examples of chemical transformations being used to modify nucleic acid residues, click chemistry is becoming ubiquitous in its ability to modify these three main areas with relative ease. 

According to the literature, most click-mediated modifications are performed on the nitrogenous bases by introducing novel base analogues [30,31,32,33], attaching fluorophores or isotopic elements for molecular imaging [34,35,36,37], forming inter-strand linkages between oligonucleotides [38,39,40], and the bioconjugation of molecules used for the assisted delivery of nucleic acid-based therapies [41]. There are, however, fewer studies focused on altering the native backbone due to the complexity and multi-step fashion of the proposed synthetic strategies and the reactivity of certain alterations to the automated process of synthesizing designer oligonucleotides [42]. Click chemistry has given researchers the opportunity to target the backbone using a fairly simple synthetic procedure, with the goal of negating the effects imposed on nucleic acid derivatives which stem from the problematic backbone. Knowing that the polyanionic structure of the native phosphodiester linkage is one of the main problems faced by oligonucleotides trying to emerge on the market as therapeutic agents [17,19,23], click chemistry can introduce a neutrally-charged and physiologically stable heterocycle at this site. That being said, most modifications performed on all three key areas of nucleic acids are done so in order to increase the bioavailability of nucleic acid-based therapies and to reduce their toxicity, all while maintaining their potency.

In this review we will be documenting the development of click-modified nucleic acid derivatives and their applications in both biological, and non-biological systems. The literature chosen for this review succeeds what was extensively covered by Amblard and colleagues in 2009 [2] and serves to compliment the review by El-Sagheer and Brown in 2010 [8] with current and novel triazole-modified ribo- and 2′-deoxyribonucleic acid compounds.

## 2. Click Modification of Nucleic Acids

### 2.1. Nitrogenous Bases

Nucleobases are the most frequently modified portions of nucleic acids due to the presence of easily modifiable sites, shorter synthetic routes for their modification, and the functional versatility of the derivatized products [36,43]. In the form of nucleotide phosphoramidite building blocks typically used to chemically synthesize DNA and RNA oligonucleotides through an automated process [44,45], nucleobase analogues can be site-specifically incorporated throughout the oligomer. Similarly, nucleotide sugar or backbone modifications can be incorporated into synthetic oligonucleotides using this process. However, unlike certain modified sugars or phosphates, all modified nucleobases have the advantage of unrestricted positioning throughout growing polynucleotides [46]. In addition, modified nucleobases can influence the major or minor grooves within DNA and RNA duplexes [47,48] while maintaining direct Watson-Crick interactions [43,49]. Certain nucleobase-modified DNA and RNA-based probes have been reported to hybridize to their targets with increased accuracy and mismatch discrimination [32,35]. Furthermore, the potency of oligonucleotides designed for gene-silencing applications can be controlled by manipulating their base-pairing thermodynamics [50]. Since the advent of the CuAAC, modifying the positions typically chosen on the heterocycles of natural nucleobases has been significantly streamlined. The most common accessible sites for chemical alteration on the purine heterocycles include, but are not limited to the 7-aza or deaza and the *N*^9^-positions of both adenine and guanine, the 6-*N* exocyclic amine of guanine and the 2-*N* exocyclic amine of adenine. Regarding the pyrimidine heterocycles, positions C5, the 4-*N* exocyclic amine of cytosine and the *N*^1^-positions are most notable for chemical modification. Aside from modifying the natural nucleobases themselves, triazoles have been introduced as 1′-furanose sugar-base tethers [51], or used for the production of synthetic nucleobase analogues as mentioned earlier. In contrast, many nucleobase modifications have been positioned at the C5 position for pyrimidines, and the 7-deaza position for purines to avoid the Watson-Crick base-pairing region and occupy space within the major groove of the DNA/RNA duplex. This strategy provides an avenue through which to click-conjugate large molecules for the purposes of delivery, fluorescent visualization, and for duplex conformational studies [37,52,53]. In addition to these functions, these base analogues also form the basis of potential antiviral and anticancer agents [54,55]. Current examples of antiviral nucleic acid-based derivatives which inspire the continued implementation of the CuAAC in developing prospective therapies include the discovery of ribavirin [25,56], a successful 1,2,4-triazole-based antiviral nucleoside analogue, and the potent anti-HIV agent known as AZT (3′-azido-3′-deoxythymidine) [57].

#### 2.1.1. ASOs and siRNAs

ASOs are single-stranded DNA (ssDNA) molecules that target and hybridize through Watson-Crick complimentarity to specific messenger RNA (mRNA) transcripts in cells in order to down-regulate the expression of problematic proteins [58,59,60]. Upon binding to the target RNA sequence, the ASO:RNA duplex either recruits RNase H which actively cleaves the RNA transcript, or physically inhibits its proper translation. In order to evaluate the efficacy of ASO-based constructs, there is much focus on increasing the thermostability of DNA:RNA interactions, while increasing the ASO’s resistance to enzymatic degradation [61]. In an effort to enhance this DNA:RNA duplex thermostability, Nielsen and colleagues chose to incorporate hydrophobic and aromatic moieties at the C5-position of pyrimidines through a triazole tether [62]. This and other studies have shown that incorporation of hydrophobic groups within the major groove can increase the stability of duplex oligonucleotides through stacking interactions [41,63,64,65]. Triazoles themselves are well suited for increasing duplex stability because they can stack favorably [66,67] and can potentially form H-bonds with the C5-proton [62].

**Scheme 1:**
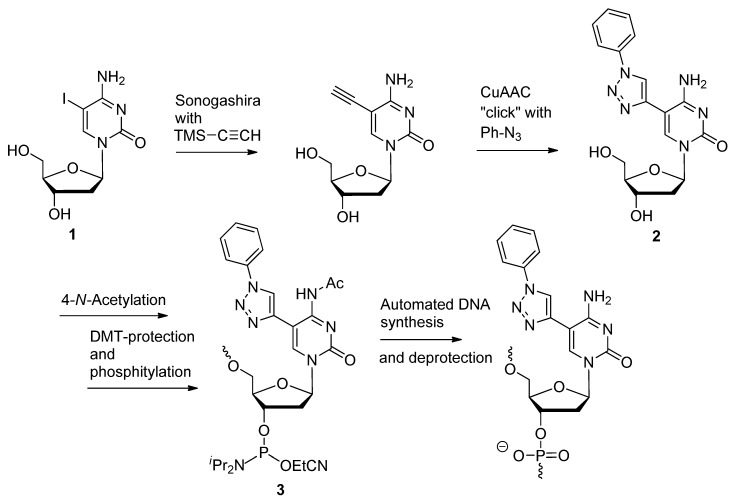
Synthesis of C5-triazolo modified pyrimidines with aromatic substituents for increased duplex stability.

**Figure 1:**
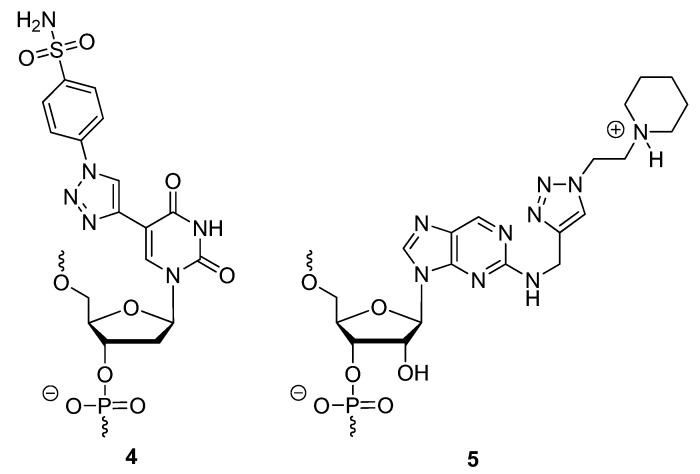
Example compounds used for ASO and siRNA-based applications.

The C5-iodo cytidine **1** [68] (Scheme 1) building block underwent a Sonogashira cross-coupling with trimethylsilane (TMS)-acetylene, which was subsequently click-cyclized with phenyl-azide to produce cytidine analogue **2** in 80% yield. After taking the appropriate steps to achieve the corresponding phosphoramidite building block **3**, several DNA sequences were synthesized containing the cytidine analogue, along with others containing the corresponding 5-(phenyltriazole)uridine [66] nucleoside. After comparing the thermostabilities of the various duplexes containing the 5-(phenyltriazole)pyrimidines, it was discovered that the uridine analogue was more well tolerated when complimented with RNA or with duplex DNA as a triplex-forming oligonucleotide (TFO). In a similar study, DNA molecules containing 5-triazole-benzenesulfonamide-modified pyrimidines such as compound **4** (Figure 1) in the central region were also found to strongly hybridize to RNA [65]. In both studies, it was observed that duplex stability increased with incremental additions of consecutive C5-triazole-modified pyrimidines in the oligomer as a result of enhanced stacking interactions.

#### 2.1.2. Oligonucleotide Based Nano-Materials

**Scheme 2:**
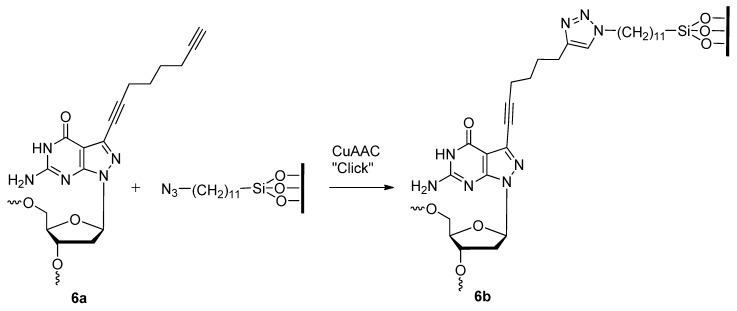
DNA strands containing 7-deaza-monoacetylenes “clicked” on to surface of azide-functionalized silicon wafers.

**Figure 2:**
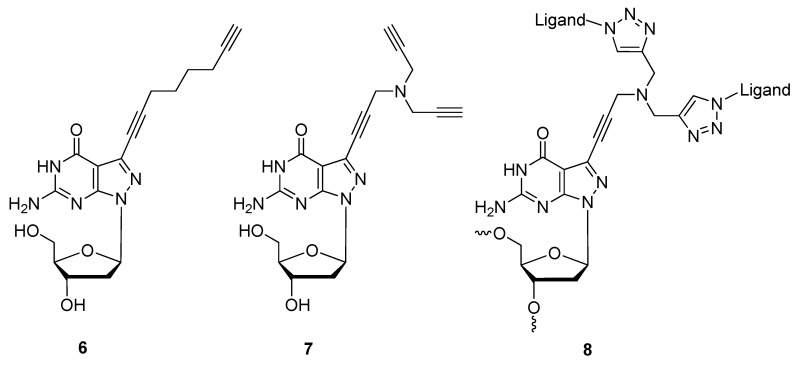
Structures of 8-aza-7-deazaguanosine analogues with 7-deaza-mono- or di-acetylenes for further click conjugation.

Finding a facile method of synthesizing new materials is of great interest to material chemists. The CuAAC offers an ideal synthetic strategy towards the development of material libraries used for various downstream applications. Some applications of triazole-based DNA nano-materials that have been explored by members of Seela’s group include click-conjugation for drug delivery [41] and the development of DNA nanopatterns on a solid support [73]. Based on the pyrazolo[3,4-*d*]pyrimidine [74] scaffold instead of a purine, they functionalized the 7-deaza position of the 2′-deoxyguanosine analogue with alkyl linkers of varying length containing terminal mono- or di-acetylenes (compounds **6** or **7**; Figure 2) [41]. After incorporating the 8-aza-7-deaza-7-(octa-1,7-diynyl)-modified 2′-deoxyguanosine (dG) analogue nucleoside phosphoramidite **6** at the 5′ end or within the central region of synthetic oligonucleotides (**6a**, Scheme 2), the modified oligonucleotides were click-ligated to azide-functionalized silicon wafers **6b** [73].

SiRNAs are double-stranded RNA molecules approximately 21 base pairs (bp) in length, whereby the active RNA strand (guide) utilizes an endogenous pathway to selectively target and cleave mRNA transcripts [18]. In addition to enhancing their target specificity or decreasing their susceptibility to enzymatic degradation [9,69], siRNAs are often chemically modified to reduce their binding to proteins which are not part of the RNA interference (RNAi) pathway [70]. Proteins containing motifs which bind to double-stranded RNAs in a sequence-dependent manner interact with these duplexes primarily through their minor groove [71,72]. Based on this knowledge, a minor-groove modification on the RNA duplex was performed by Beal and colleagues in which a 2-*N*-(methyltriazolyl)ethylpiperidine purine analogue **5** (Figure 1) was substituted into both the non-active passenger and guide strands to influence the protein-binding properties of these RNAs [47]. Not only did the resultant RNAs modified with this compound retain their gene-silencing activity, but those that were modified in the non-active passenger strand exhibited reduced binding to the adenine deaminase ADAR1 (adenine deaminase that acts on RNA-1).

The immobilized oligonucleotides formed self-assembly patterns due to the position of the dG analogue and compounded intra- and intermolecular forces such as phosphodiester-nucleobase interactions, base [75] and triazole [66] stacking interactions, and the hydrophobicity of the alkyl linkages. Seela and colleagues continued to functionalize compound **7** (Figure 2) through the double-click cycloaddition of various azides such as benzyl-N_3_, PEG-N_3_, AZT, and a C_11_-N_3_ alcohol for the purposes of drug delivery (**8**, Figure 2) [41].

#### 2.1.3. Structural Determination

**Scheme 3:**
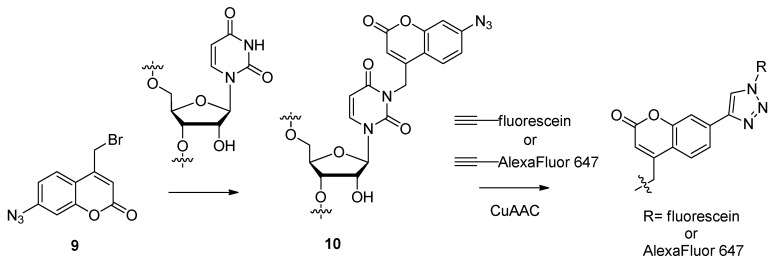
Site-specific *N*^3^-alkylations of uridine residues with the coumarin-based compound N3BC.

Many functional groups are being used as potential molecular labels for further elucidating the structures and functions of DNA and RNA-based compounds. Rather than site-specifically incorporating a pre-functionalized nucleoside into synthetic oligonucleotides, Helm and colleagues developed a coumarin chromophore-based post-synthetic RNA alkylating agent **9** they labeled as N3BC (Scheme 3) [76].

**Scheme 4:**
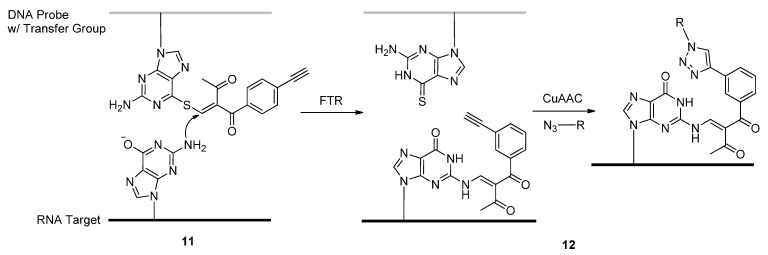
Template-driven central modifications of target RNA sequences through a functionality-transfer reaction (FTR).

N3BC, which was synthesized in five steps starting from 3-aminophenol for an overall yield of 44%, was found to be specific for alkylating the *N*^3^-position of uridines (**10**, Scheme 3) once exposed to the target RNA sequence and has a coumarin-based excitation wavelength of 320 nm. Alternatively, the N3BC-modified RNAs could undergo conjugation with alkyne-functionalized fluorophores or other ligands due to the free azide moiety. An alternative method of post-synthetically modifying the central region of native RNA molecules was developed by Sasaki and colleagues in which a functionality-transfer reaction (FTR) takes place between the DNA-based probe carrying the desired modification and the complimentary RNA sequence destined for modification (Scheme 4) [77]. After incorporating C6-thioguanosine into the synthetic DNA probe, the C6-thio is targeted for alkylation with the 2-methyliden-1,3-diketone transfer group. After the probe hybridizes with the complimentary target RNA sequence, the FTR selectively proceeds to modify the C2-amino group of the native guanine base opposite from the functionalized C6-thioguanosine (**11**, Scheme 4). The FTR-modified RNA molecule containing a terminal alkyne from the transfer group could undergo further click-mediated ligation with various azide compounds (**12**, Scheme 4). The initial results from these click ligations showed nearly quantitative yields with all compounds tested [77].

**Scheme 5:**
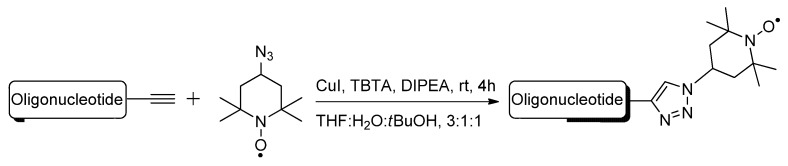
The post-synthetic click-ligation of the 4-azido-TEMPO spin label to alkyne-functionalized oligonucleotides.

**Figure 3:**
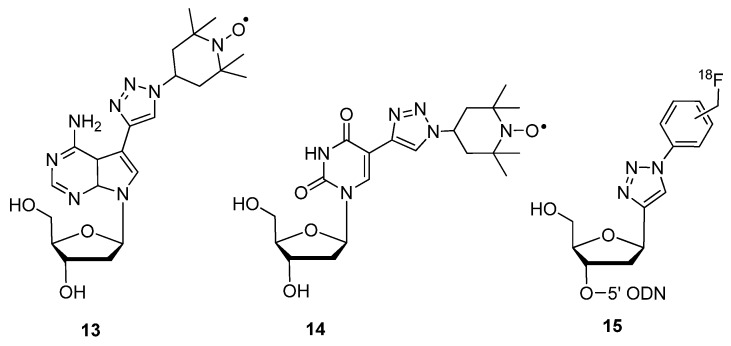
Structures of purine, pyrimidine or nucleobase mimics containing triazole-tethered spin-labels.

To examine the folded structures and conformation of DNA or RNA oligomers within biological environments, electron paramagnetic resonance (EPR) [78] coupled with site-specific spin-labeling of the oligomers has become a useful tool. In preparation for use of this technology, the Seela group used click chemistry to modify DNA and RNA molecules containing alkyne-functionalized purine and pyrimidine analogues **13** and **14** (Figure 3) with the radical azide nitroxide 4-azido-2,2,6,6,-tetramethylpiperidine 1-oxyl (4-azido-TEMPO) [79] spin-label (Scheme 5) [52]. This was the first example of click-modifying oligonucleotides at the site of the nucleobase with spin-labels for subsequent EPR analysis.

Positron emission tomography (PET) is another molecular imaging technique which can track the distribution and behavior of radiolabeled bioactive molecules *in vivo* [80]. Most PET scanning procedures involve the use of ^18^F-radiolabeling due to its short half-life and covalent stability [81]. The 1,3-dipolar click cycloaddition has also been employed as a quick and efficient method of attaching nucleobase mimics to the 1′-position of the furanose ring. Utilizing this efficient strategy to modify nucleoside furanose rings, compound **15** (Figure 3) containing the ^18^F-radiolabel was synthesized, starting with the 1′-*β*-ethynyl-2′-deoxyribonucleoside building block and its terminal incorporation into synthetic oligonucleotides via phosphoramidite chemistry [82]. The meta- or para-radiolabeled 1-azido-4-([^18^F]fluoromethyl)benzene prosthetic group was then post-synthetically click-conjugated to the 1′-ethynyl building block within the modified oligonucleotide.

#### 2.1.4. Fluorescent Labeling

Attaching fluorescent labels to nucleobases [35,37,53] or making the bases an integral part of the fluorophore [36,46,83] is a highly sought after practice for the labeling of oligonucleotides. Since fluorescent nucleobases in DNA and RNA oligonucleotides hybridize directly to the target oligonucleotide or protein, they can provide visual representations of oligo-target hybridization properties as probing tools [35] or can gather information to form structure-activity relationships [76]. The ease of attaching either fluorescent or fluorescence-promoting compounds to nucleobases via the CuAAC has resulted in numerous reports in this field. It is also worth noting that fluorescence can be triggered once a triazole moiety is appended to certain nucleobases, such as adenosine at the C8 position [84,85].

**Figure 4:**
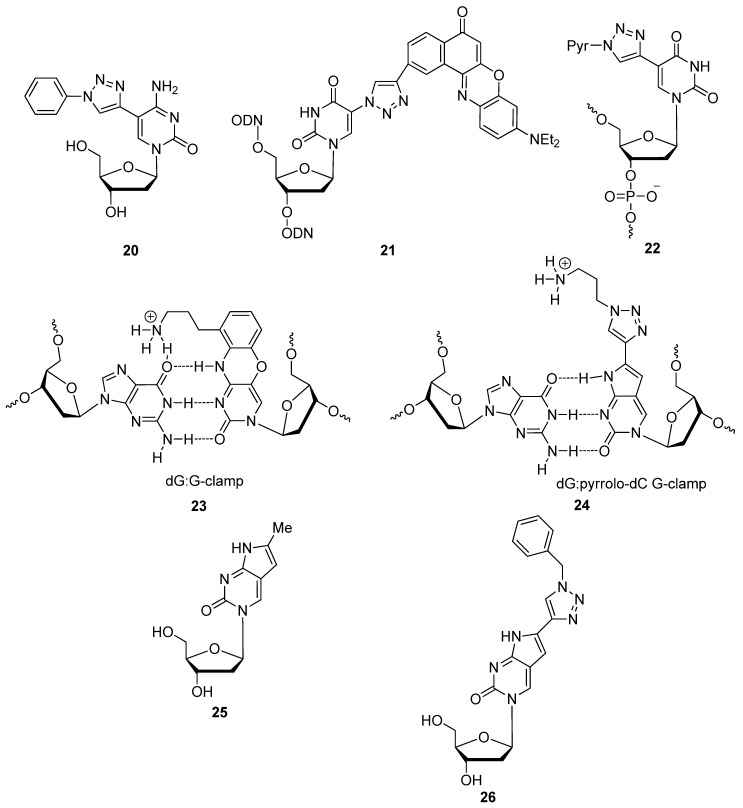
Triazole-modified nucleic acid-based compounds with fluorescent properties which also serve as precursors for other applications.

Although we have already discussed other uses for C5-triazole-modified pyrimidines, the following report describes the synthesis of the fluorescent C5-triazolylcytidine nucleoside analogue **20** (Figure 4). This cytidine analogue was the first example of a C5-ethynylcytidine building block being click-cyclized with an azide residue to form a fluorophore, without the attachment of an actual fluorophore to the molecule [36]. As previously outlined, a motivation for modifying the C5 position of pyrimidines is that these modifications are well accommodated within the major groove of duplex oligonucleotides, thus leaving the overall natural conformation of the duplex unperturbed. In the same year, a similar study click-conjugated the fluorophore Nile Red at the C5-position of uridine to yield compound **21** (Figure 4) [53]. The difference with the synthetic scheme presented in the latter study was that the C5-iodopyrimidine was first incorporated into oligonucleotides, followed by a post-synthetic azide displacement at the C5 with NaN_3_ and the click-attachment of the alkyne-functionalized dye. Using fluorescence to detect the presence of single-nucleotide polymorphisms (SNPs) in target DNA or RNA sequences, Hrdlicka and colleagues used the CuAAC to attach a pyrene residue at the C5 position of uridine through a triazole moiety [35]. Compound **22** (Figure 4) showed favorable mismatch discrimination by significantly decreasing its quantum yield in the presence of mismatched sequences.

**Scheme 6:**
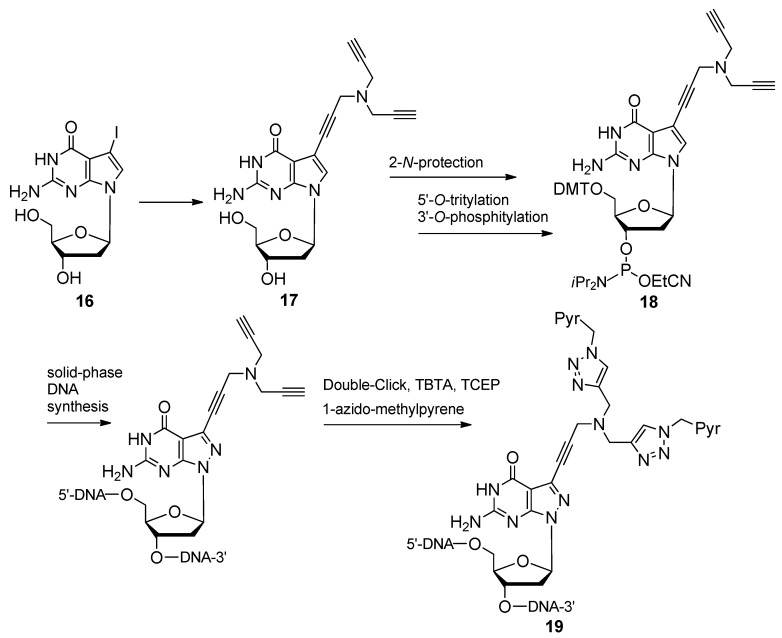
Synthesis and oligonucleotide incorporation of double-click conjugated bis-pyrene 2′-deoxyguanosine analogues.

The expansion of the pyrene′s application as a fluorescent compound is made evident when 1-azidomethyl pyrene is “double-clicked” with the reaction substrate 7-tripropargylamine-7-deaza-2′-deoxyguanosine containing two terminal acetylenes (Scheme 6) [37]. These labeled guanosine analogues differed slightly from the 8-aza variant of the compound **7** previously mentioned by the Seela group (Figure 2). The starting material 7-iodo-7-deaza-2′-deoxyguanosine **16** [86] (Scheme 6) underwent the Sonogashira cross-coupling reaction with a ten-fold excess of tripropargylamine, producing the aforementioned di-acetylene **17** on the 7-deaza position. After transforming the di-acetylene into the appropriate phosphoramidite **18**, this compound was used to site-specifically incorporate the bis-pyrene modification at various positions within 12-mers of ss- and dsDNA as a dG substitution. The melting temperatures (*T_m_*s) of all di-acetylene-modified oligomers were slightly increased above the reference value (50 °C) ranging from 50–56 °C, indicating that the larger tripropargylamine linkages did not disturb the native duplex structure. After double-clicking the 1-azidomethyl pyrene residues to the di-acetylenes (**19**, Scheme 6) and in addition to the noticeable increase in duplex *T_m_*s, excimer fluorescence [87] was achieved between bis-pyrene-modified purines when placed on opposite strands of the duplex and separated by two base pairs.

Seela and colleagues have adopted the CuAAC as the single-most useful tool for performing facile transformations on nucleosides to produce novel building blocks which find use in a variety of applications. Although not related to fluorescent labeling, this report by Seela’s group synthesized the triazole-modified compound **24**, to be a mimic of the G-clamp [88] (Figure 4). The G-clamp **23** is a nucleoside analogue which is known to provide greater stability to 2′-deoxyribocytidine:guanidine (dC:dG) interactions when substituting dC residues [89]. Their goal was to fortify the duplex stability within parallel-stranded DNA (psDNA) through this alternative G-clamp mimic, in order to explore the possibilities presented by psDNA as a future biomimetic for biological applications. Adopting the design of this G-clamp mimic **24** which uses the pyrrolo-dC [83] scaffold **25** and its native fluorescence capabilities, Seela’s group synthesized an alternative triazole-modified pyrrolo-dC derivative **26**, displaying a quantum yield ten times greater than the reference design [32]. In contrast to the results presented by Hrdlicka and colleagues regarding the uridine analogue **22** with a C5-triazolo-pyrene and its mode of action for detecting mismatches [35], compound **26** experiences severe quenching of fluorescence when hybridized to matching sequences and an increase in quantum yield as mismatches occur. Since derivative **26** represents a substitution for dC, the compound′s fluorescence is most severely quenched when opposite a dG residue on the target sequence. The quantum yield slowly increases in the following order depending on the opposing residue: dG < dT < dA < dC.

#### 2.1.5. Anticancer and Antiviral Development

The discovery of antiviral agents such as AZT and Ribavirin have inspired chemists to investigate the use of the CuAAC to develop libraries of potential antiviral and anticancer therapeutics [90]. With the knowledge that azides and alkynes are selectively reactive with each other, are inert moieties when exposed to biological conditions, and cyclize to produce a stable 1,4-disubstituted isomer of the 1,2,3-triazole, the CuAAC has shown great versatility in producing numerous nucleic acid-based derivatives which target molecules implicated in the propagation of viral disease and cancer [1,3,4,8].

**Figure 5:**
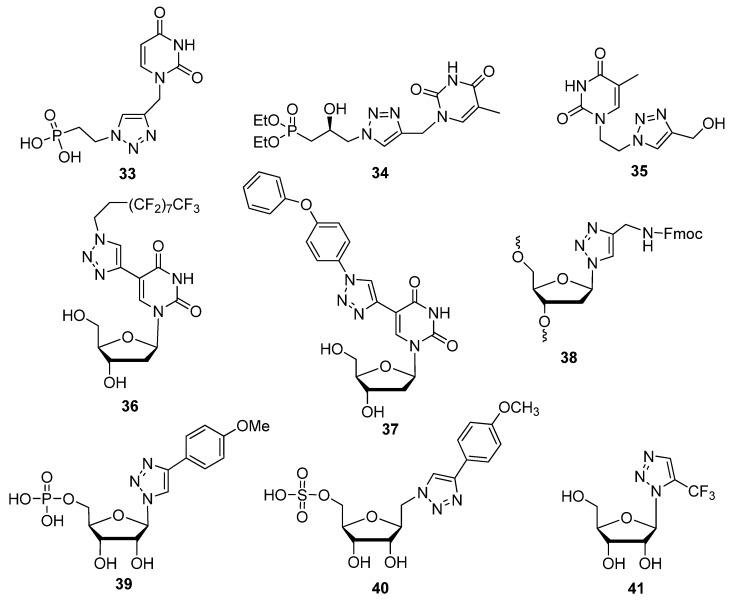
Nucleic acid-based compounds displaying anticancer or antiviral properties.

Mono-nucleoside compounds which have proven themselves to be useful anti-pathogenic agents are acyclic nucleoside phosphonates (ANPs) [91]. In place of the typical furanose ring found in native nucleotides is an acyclic alkyl chain connecting a phosphonate moiety to the *N*^1^-position of pyrimidines or the *N*^9^-position of purines. Some examples of click-modified ANPs are compounds **33** [22] and **34** [92] (Figure 5) which showed potential against the Hepatitis C Virus (HCV) [22] or as a future antiviral agent [92]. An *N*^1^-acyclic pyrimidine modification which did not include a phosphonate moiety is compound **35**, designed for its intended use as an antivirulent and showing promising preliminary results as an antioxidant [93].

Compounds such as 5′-fluorouracil (5′-FU) [94], floxuridine, gemcitabine and clofarabine are examples of known fluoronucleoside-based anticancer compounds. The effectiveness of these compounds is in some cases the driving force behind the rapid click-mediated production of fluorinated compound libraries with anticancer potential [54]. Using the same synthetic strategy to modify the C5 position of 2′-deoxypyrimidines [36,62], all C5-modified pyrimidine analogues containing a perfluorodecyl-substituted triazole unit such as derivative **36** showed inhibition of cancer cell growth [54]. Agrofoglio and colleagues also made a contribution after designing a library of C5-substituted click adducts upon discovering that compound **37** not only inhibited a variety of DNA-based viruses, but also displayed anti-tumor activity which was comparable to the reference compound 5′-FU [55].

**Scheme 7:**
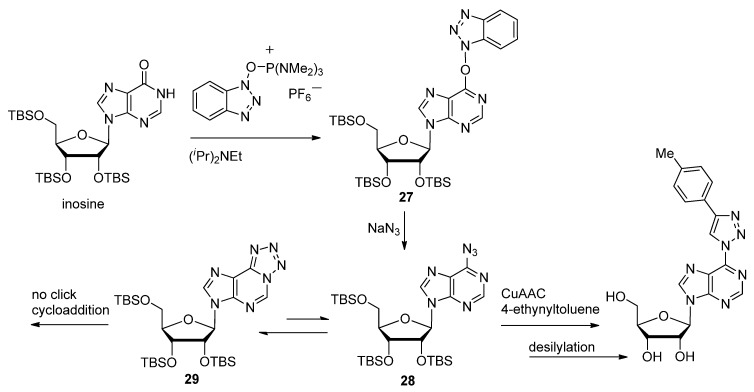
Synthetic strategy to isolate the C6-azido purine tautomer for click-cyclization with various alkynes.

Lakshman and coworkers modified the *O*^6^-position of inosine with an azide group, in order to form a click attachment point for several alkyne-modified functionalities such as 4-ethynyltoluene (Scheme 7) [95].

**Scheme 8:**
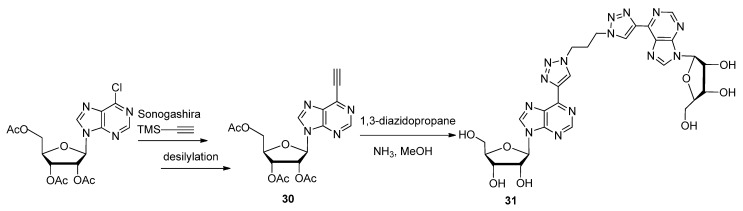
Synthesis of triazole-linked di-nucleosides to be used as anti-adenosine receptor ligands.

After the azide displacement reaction with the protected *O*^6^-(benzotriazol-1-yl) intermediate **27**, the azide **28** would equilibrate with its *N*^1^-tetrazolyl tautomer **29**. Until solvent conditions were optimized to isolate the azido tautomer as the major reactant, the CuAAC conjugation of alkyne-based functionalities was low-yielding or did not occur. Realizing that formation of the tetrazolyl tautomer was a potential hindrance to the CuAAC, Mathew *et al*. decided to use a C6-chloro-protected purine as the substrate for the Sonogashira cross-coupling reaction with TMS-acetylene to introduce a C6-acetylene (**30**, Scheme 8) [96]. The result of this alternative synthetic strategy coupled with the CuAAC lead to the discovery of several different triazole-modified derivatives such as the homo-purine dimer compound **31**. Both articles focused on the development of agonists or antagonists for adenosine receptors, since they are expressed in almost every cell and therefore present a popular target for potential drugs [96].

**Scheme 9:**
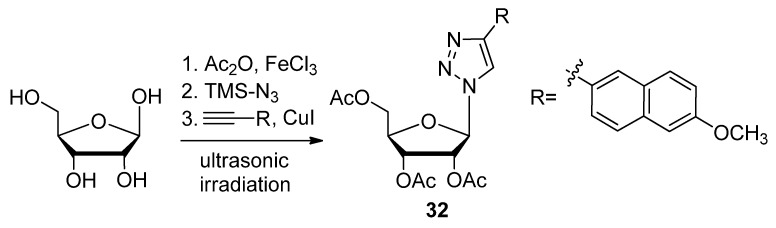
One-pot synthesis of C1′-triazolo nucleobase mimics with ultrasonic irradiation.

Referring back to triazole-based substitutions at the 1′-furanose position, there are a few examples of bioactive mimics containing functionalities attached through a five-membered ring systems at this site [97]. Capitalizing on this trend, the following Fmoc-protected 4-(aminomethyl)-1,2,3-triazolyl nucleoside analogue **38** (Figure 5) was synthesized for its potential use as an antiviral agent [98]. A more recent example of a 1′-triazole-modified nucleoside analogue which could have antiviral effects is compound **39**, containing a 4-(triazolyl)-*p*-methoxyphenyl substituent which successfully inhibited an *E. coli* nicotinamide adenine dinucleotide malic enzyme (NAD ME) [99]. An expedient method of implementing the click-modification of a nucleoside mimic at the 1′-furanose position was developed with the assistance of ultrasonic irradiation [97]. With the help of an Fe(III) catalyst (FeCl_3_), this one-pot synthetic strategy to click-modify a 1′-azidofuranose building block using ultrasonic irradiation reduced reaction times from hours to minutes while increasing the overall yield (Scheme 9). 

In addition, the active compound **32** significantly reduced the viability of chronic myelogenous leukemia (CML) K562 cells by 93%, compared with a known CML inhibitor AICAR which lowered cell viability by 35%. Itzstein and colleagues further extended the click-based modification at the 1′-sugar position by adding a methylene bridge at the sugar-triazole junction [100]. The resultant 1′-homo-*N*-nucleoside derivative **40** (Figure 5) will be tested for its application as an antivirulent. Another group used a unique TBS-directed CuAAC method to force the production of the 1,5-disubstituted regioisomer of the more common 1,4-1,2,3-triazole [33]. Their inspiration came from examples of fluorinated and highly-bioactive molecules containing 1,5-1,2,3-triazoles [101]. The antiviral and cytotoxic evaluation of compound **41** with its 5-trifluoromethyl substituted 1,2,3-triazole in the 1′-furanose position is underway.

### 2.2. Sugar Modifications

Modifications at the sugar moiety of nucleosides are commonly employed for the purpose of developing novel anti-viral or cancer therapeutics and for applications which would benefit from appending useful molecules to mono- or oligonucleotides. Applications of nucleoside analogues with modified sugars are generally the same as for analogues with modified bases and backbone structures. However, the sugar moiety is restricted to a small number of modifiable sites. The positions which are modified on the sugar are typically C1′ to C5′ (or C6′ for locked nucleic acids (LNA) [102]) [48]. For the purposes of this review, we will be focusing on the sugar positions C2′, C3′ and C5′. Nucleoside analogues where modifications are performed on the C3′ or C5′ positions are either meant to be used as monomer or dimer units, or occupy the terminal ends of DNA or RNA oligonucleotides [57,103,104,105,106,107]. The C2′ position offers the opportunity to place modified nucleoside analogues throughout chemically synthesized oligonucleotides since elongation usually occurs from the C3′ or C5′ positions [48,108,109,110]. The CuAAC has found utility in attaching molecules to these positions through a triazole tether, especially since AZT is a readily available starting material containing a C3′-azido group [57]. Using common procedures to functionalize these sugar positions with either azides or alkynes, the CuAAC has become a great tool in churning out libraries of potentially useful nucleic acid-based compounds.

#### 2.2.1. C3′-Modified Sugars

**Figure 6:**
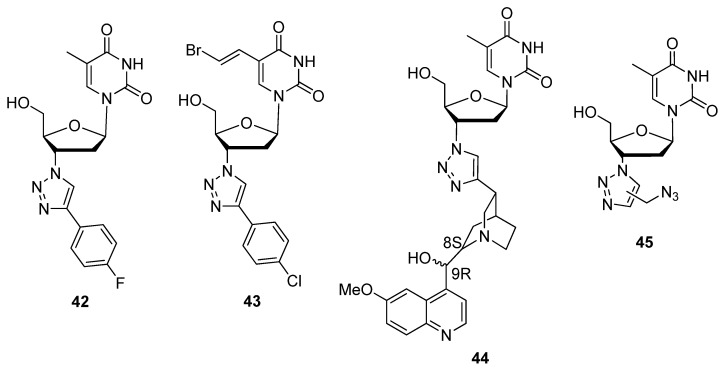
Various C3′-triazole modified 2′-deoxyfuranoses.

Many researchers utilize the CuAAC with AZT as the precursor thymine analogue in order to functionalize the C3′ using a variety of alkynes for various applications. For C3′-triazolo-modified compounds synthesized from AZT as the precursor, refer to Figure 6. The goal for the first two structures which were derivatized from AZT was to act as inhibitors of 2′-deoxynucleoside kinases (dNKs) such as thymidine kinases in particular. Thymidine kinase 1 (TK1) was the target of compound **42** (Figure 6), among several compounds synthesized from AZT by Erikkson and colleagues [57]. TK1 is responsible for the rate-limiting step of initiating DNA replication [111] and is over-expressed in malignant cell lines [112], thus making it a suitable target for synthetic ligands. Van Calenbergh and colleagues synthesized nucleoside analogues with an additional C5-(2-bromovinyl) group on the nucleobase [103]. An example is compound **43** which was selective for thymidine kinase 2 (TK2), a dNK which is responsible for phosphorylating dNTPs for mitochondrial DNA replication [113]. Their goal was to understand the mechanism through which nucleic acid-based antiviral or anticancer therapies cause mitochondrial DNA toxicity. Compound **44** was synthesized to serve as an anticancer agent and as an optional fluorescent tag at the 3′-ends of oligonucleotides due to the quinine substituent [105]. With the intention of producing inhibitors for the popular drug target HIV-1 RT, Roy *et al*. modified AZT with both 1,4- and 1,5-disubstituted isomers of the azide-functionalized 1,2,3-triazole **45** based on AZT′s success at inhibiting the same enzyme [107].

#### 2.2.2. C5′-Modified Sugars

**Figure 7:**
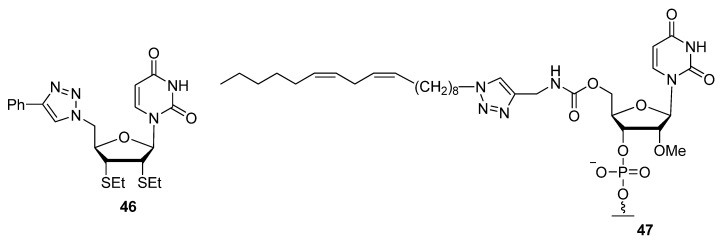
Uridine nucleoside analogues C5′-triazole modified sugars.

Ribose sugars with C5′ azide or alkyne groups can be introduced at the 5′-ends of synthetic oligonucleotides and along with many other applications, they can be further click-functionalized with molecules to improve delivery [106] or to enhance their specificity and potency as cytotoxic agents [104]. In the pursuit of therapeutic design, Wu and colleagues explored a series of C5′-triazolo modified uridines, thymidines and cytidines to screen for anticancer properties [104]. Compound **46** (Figure 7) showed substantial cytotoxic profiles against three of the four cancer cells lines tested, surpassing the reference compound 5′-FU with a six-fold increase in potency. With an interest in gene-silencing therapies, Manoharan and colleagues attached long-chain fatty acids to alkyne-functionalized sugars throughout synthetic siRNAs. They discovered that siRNAs containing compound **47** at the 5′ end of the sense strand displayed enhanced activity compared to all other modified constructs, including the wild-type duplex [106].

#### 2.2.3. C2′-Modified Sugars

**Figure 8:**
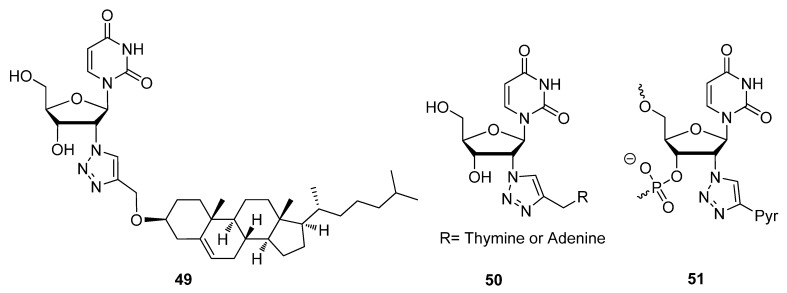
Structures of C2′-triazole modified nucleosides.

The last optional position to modify at the furanose sugar moiety in nucleic acid residues while remaining within the scope of this review is the C2′ position. Unlike the C3′ and C5′ positions, nucleic acids containing C2′ sugar modifications can still be incorporated throughout synthetic oligonucleotides since this position does not typically participate in strand elongation. Towards the development of C2′ modified nucleoside monomers, Liebscher and colleagues devised a method of click-appending cholesterol molecules with variable-length aliphatic linkers to the C2′ position of uridine [109]. Using various solid-state and 2D-NMR spectroscopic techniques, this group verified that compound **49** (Figure 8) containing a cholesterol group tethered to the C2′ sugar position through a triazole moiety could anchor to 1-palmitoyl-2-oleoyl-*sn*-*glycero*-3-phosphocholine (POPC) membranes. Capitalizing on the successful design of double-headed nucleosides for potential therapeutic applications or for DNA and RNA structural studies [114], another group extended the design by attaching nucleobases to various positions of the sugar moiety, particularly the C2′ position [48]. Incorporating compounds **50** into duplex DNA or RNA molecules, Nielsen and colleagues studied the effects of these double-headed derivatives on nucleic acid secondary structures.

**Scheme 10:**
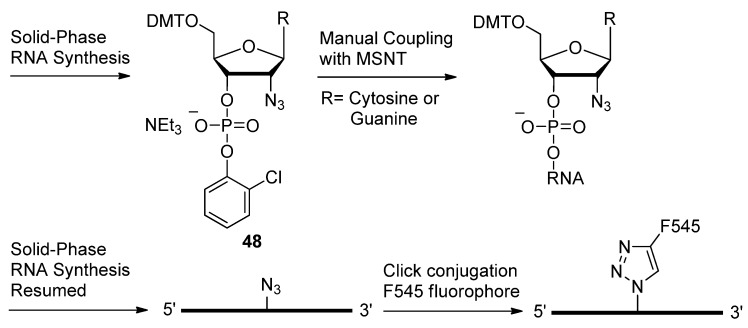
RNA synthesis using phosphoramidite chemistry in the presence of free azide moieties and their compatibility with the chemistry.

Based on their previous work with 2′-azido modified 2′-deoxyuridine/adenosine analogues which were compatible with siRNA constructs [115], Fauster *et al*. extended the work by producing cytidine and guanosine 2′-azido modified analogues [108]. The synthetic assembly of oligonucleotides using phosphoramidite chemistry has generally proven incompatible with azides. In the presence of P(III), azides have proven to be highly susceptible to the Staudinger reduction [116]. It is for this reason that most groups who wish to click-derivatize their oligonucleotides, tend to append alkyne groups to their oligomers in preparation for cyclizing with azide-functionalized molecules. Despite these findings, Fauster *et al*. went ahead to prove that azide functional groups at the furanose C2′-position are compatible with the steps of automated oligonucleotide synthesis (Scheme 10) [108].

Phosphoramidites **48** were site-specifically incorporated into the growing oligonucleotide to place the C2′ azide in the desired position(s), using 1-(mesitylene-2-sulfonyl)-3-nitro-1,2,4-triazole (MSNT) [117] as an activator. Automated addition of remaining residues continued without affecting the free azide moieties on the oligomer. The resulting azido-siRNAs were then post-synthetically labelled with the fluorophore F545 through the CuAAC. Even though pyrene is commonly used as a fluorescent label due to its innate character as a fluorophore [118], this next group used this aromatic functionality to produce non-discriminatory DNA and RNA probes [110]. These universal probes can be useful when the target sequence contains a couple of unknown residues. In the presence of mismatched sequences or abasic sites, compound **51** (Figure 8) was least discriminating as determined by the minimal perturbations in probe:target duplex *T_m_*s. The tentative explanation for this behavior is that the pyrene substituent is intercalating into the duplex core and pushing out the opposing nucleobase, thus allowing for universal hybridization devoid of a Watson-Crick interaction.

### 2.3. Backbone Modifications

The goals which stem from modifying all three key areas of nucleic acids are relatively the same, in that all studies pursue the advancement of both structural and functional characteristics of nucleic acid-based derivatives. In order to retain or to enhance properties which are favorable for ASO and siRNA-based constructs, modifications at the site of the backbone are also of great interest. Examples of areas which would benefit from backbone-targeted modifications include the production of longer or multi-branched oligonucleotides and synthetic genes. Currently, there is an upper limit of approximately 100 bases in the length of DNA by using traditional DNA and RNA synthesis techniques such as solid-phase phosphoramidite chemistry [119]. To overcome this size limitation, a new class of backbone-modified oligonucleotides that utilize the 1,4-disubstituted 1,2,3-triazole as the internucleotide linkage has emerged in the last decade [26,120]. Another attractive feature in utilizing triazole-linked oligonucleotides is the enhanced resistance towards endo- and exonuclease-mediated cleavage, which improves construct bioavailability within physiological environments [2,8,19].

#### 2.3.1. DNA Backbone Modification

**Scheme 11:**
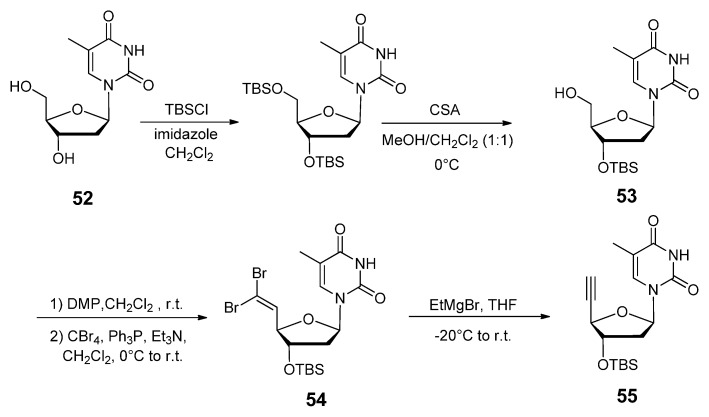
Synthesis of the thymidine 4′-alkynylated monomer.

**Figure 9:**
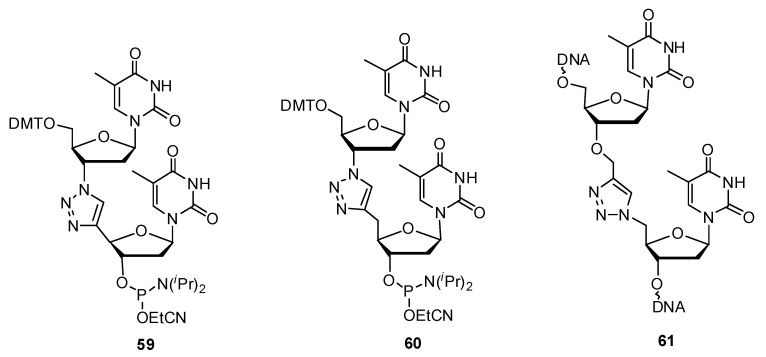
DNA oligonucleotides containing triazole-based internucleotide linkages.

In one study by Chandrasekhar *et al*., a triazole linked 2′-deoxythymidine nucleoside phosphoramidite **59** (Figure 9) was synthesized for its direct use in solid-phase oligonucleotide synthesis [121]. This dimer unit can be placed at any requisite site in the chain. They designed a new dinucleoside phosphoramidite with the sugar part of the nucleotide unaltered. The monomer unit **55** (Scheme 11) containing the terminal acetylene moiety which serves as the precursor for the prospective triazole-linked dimer was synthesized from commercially available thymidine **52** in five steps. Succeeding the protection of free alcohols, the group then selectively deprotected the 5′-OH with 10-camphorsulfonic acid [122], yielding derivative **53**. The free alcohol was then oxidized with Dess-Martin periodinane, subjected to the Corey-Fuchs protocol [123] and then treated with ethylmagnesium bromide (**54**, Scheme 11). The two key fragments which were the azide unit (AZT) and the terminal acetylene **55** were then clicked together through the CuAAC reaction to yield the target triazole-linked dimer phosphoramidite **59** (Figure 9).

Sequentially heterogeneous oligonucleotides bearing a similar triazole-linked dimer **60** (Figure 9) was synthesized by the Florentiev group [124]. Their triazole-based linker was one atom longer than Chandrasekhar’s modification. Similarly, their synthetic strategy also included the use of Dess-Martin periodinane in order to oxidize the 5′-OH to an aldehyde, after which the dibromoalkene was synthesized as an intermediate to convert the aldehyde into a terminal alkyne. The CuAAC reaction was then carried out between the terminal alkyne and 3′-azido-3′-deoxy-5′-*O*-dimethoxytritylthymidine. After assessing the effects of single and multiple modifications on the thermostability of mixed-base duplexes, an average drop in *T_m_* of −4 °C was observed indicating that the incorporation of this triazole linked dimer was slightly destabilizing for its hybridization ability.

**Scheme 12:**
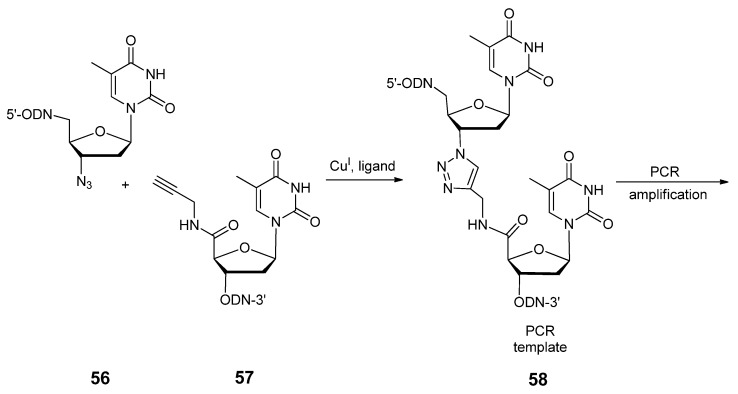
Synthesis and PCR amplification of an unnatural triazole-based DNA backbone.

A click-ligated DNA strand **58** (Scheme 12) made up of 81 residues has been synthesized between oligonucleotides containing 3′-AZT **56** and 5′-propargylamido-2′-deoxythymidine **57** residues [125]. In order to more efficiently assemble the oligonucleotides with 3′-AZT and avoid the potential incompatibility of the azide group with P(III) chemistry [116], the required sequences were assembled in the 5′ to 3′ direction using 3′-DMT-5′-phosphoramidites of DNA nucleotides. To produce the azide-functionalized DNA strand, AZT was added as a phosphotriester monomer to the 3′-end of the designer strand after its automated assembly had terminated. The alkyne-modified oligonucleotides to serve as partners for the 3′-AZT strands were assembled through the 5′-ended solid-phase coupling of the 5′-propargylamido thymidine analogue (**57**) phosphoramidite, which was synthesized from the carboxylic acid derivative of thymidine [126,127]. The resultant click-ligated DNA strand displayed compatibility with various polymerases as a PCR template and at the time, served as the first example of a novel DNA ligation which could undergo reproducible PCR amplification.

Brown and colleagues not only explained the biocompatibility of the triazole linkage within synthetic oligonucleotides [128], but also examined the structure and base-pair dynamics of a DNA duplex containing a single triazole linkage between two central thymidine residues (**61**, Figure 9) using NMR solution techniques [129]. The triazole-modified DNA duplex was found to share structural similarity with the reference B-form helical conformation. Although a slight disturbance in the thermodynamic profile of the modified duplex is observed, the triazole linkage **61** promises biocompatibility with polymerases or other such biomolecules.

#### 2.3.2. RNA Backbone Modifications

**Scheme 13:**
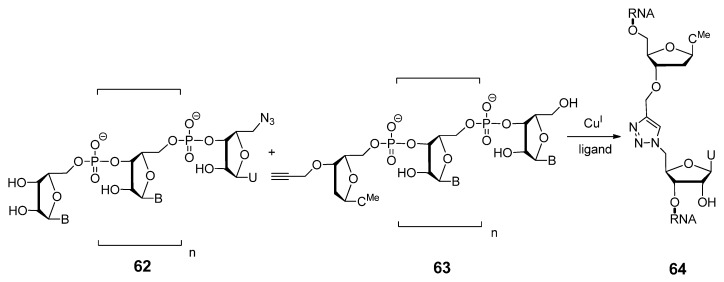
Click-mediated synthesis of the DNA-RNA hydrids.

In addition to triazole-modified backbones in DNA molecules, a number of RNA analogues have been synthesized using click chemistry, in which the native phosphodiester linkages are replaced by triazoles. Examples of such analogues were demonstrated by El-Sagheer and Brown through the click-ligation of smaller azide and alkyne functionalized ribo- and deoxyribonucleotide polymers, into a series of catalytically active RNA and DNA:RNA chimeric hairpin and hammerhead ribozymes of approximately 100 nucleotides in length [26]. After synthetically assembling and derivatizing individual oligonucleotides with either azide or alkyne functional groups, they are click-cyclized to produce larger molecules. In order to produce these RNA or DNA:RNA hydrids (**64**, Scheme 13), click-ligation occurred between terminal azides or acetylenes placed on either 3′ or 5′ ends of individual DNA or RNA strands, as displayed on the 5′-azido modified uridine and 3′-propargylated-2′-deoxycytidine analogues (**62**, **63**). Both modified hairpin and hammerhead ribozymes were shown to cleave their substrates, thus indicating the potential compatibility of these triazole-based backbone linkages with many biologically important DNA or RNA molecules. 

**Figure 10:**
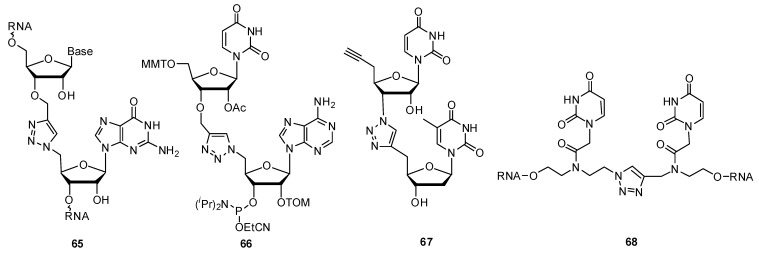
Structures of nucleoside analogues linked through a triazole moiety.

Paredes and Das extended this work by enzymatically incorporating “clickable” azido groups onto 5′ and 3′ terminal ends of an RNA oligonucleotides via novel azide-labeled nucleotide triphosphates (NTPs) [120]. Once the clickable groups were installed on individual RNA strands, rapid click radiolabeling could ensue, or template/non-template driven RNA-RNA click ligations were then possible. Linkage **65** (Figure 10) is the result of a successful template-driven RNA-RNA click ligation using fragments of the hepatitis delta virus (HDV) ribozyme. The rate and the extent of target cleavage displayed by the triazole-modified HDV ribozyme was indistinguishable from the transcribed wild-type HDV ribozyme, again indicating a broad applicability of click chemistry in RNA-comprised biological systems. 

In 2011, Rozners and colleagues synthesized a uracil-adenosine phosphoramidite **66** (Figure 10) containing a triazole backbone unit [130]. The phosphoramidite approach allows the flexibility to incorporate the desired modification in a site-specific manner throughout the growing oligonucleotide construct. The key consideration in their design was minimizing the synthetic effort and their approach involved the coupling of a 3′-*O*-propargyl uracil monomer with a 5′-azidoadenosine monomer. Reporting a largely destabilizing effect on the RNA double helix (∆*T_m_* −7 °C to −14 °C per modification in a 10 bp duplex), this group concluded that the triazole-based linkage was not an ideal replacement for the native phosphodiester linkage. In contrast to these findings and following their success with the solution-phase synthesis of unique triazole linked DNA (^TL^DNA) 8-mer in 2008 [131], Isobe and colleagues extended their efforts towards the solid-support synthesis of a triazole-linked RNA analogue **67** on a solid support through the CuAAC [132]. Proton NMR analysis of these monomeric ribonucleoside analogues illustrates a preference for the C3′-endo puckering pattern found in natural ribonucleosides, which suggests a possible use for these triazole-linked nucleoside analogues within RNAi-based applications. With the intention of utilizing novel triazole linkages dispersed throughout duplex siRNAs, our lab reported the synthesis of a nucleoside dimer analogue **68** modified at the backbone with a triazole functionality [133,134]. These unnatural triazole-modified siRNAs were capable of silencing the exogenous reporter gene *firefly* luciferase, along with the endogenous gene glyceraldehyde-3-phosphate dehydrogenase (GAPDH) in a dose-dependent manner. Incorporating these novel dimer analogues at the 3′-overhangs of siRNAs imparted an added resistance to nuclease-mediated degradation when compared with unmodified duplexes. Despite the reduced thermostability associated with these modifications, this study highlighted the first reported application of these novel triazole-based internucleotide linkages within functional siRNAs.

#### 2.3.3. Other Backbone Modifications

**Figure 11:**
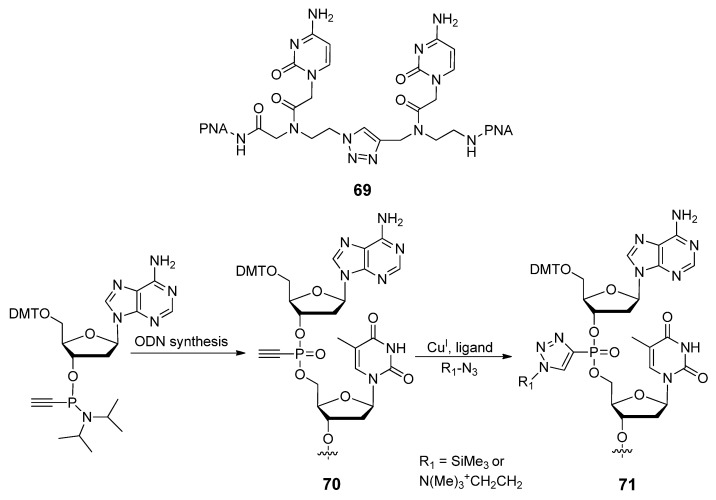
Synthesis of 1,2,3-triazolylphosphonate internucleotide (TP) linkages and the triazole-linked dimer within PNA.

The Winssinger group reported the substitution of an amide linkage in peptide nucleic acid (PNA), a successful oligonucleoside mimic made of repeating aminoethyl-glycine units [135], with a triazole linker [136]. PNA oligomers terminated with an azido monomer were prepared by standard Fmoc-based PNA synthesis [137], which were then cyclized with various alkyne monomers by means of the CuAAC reaction. The target PNA-based dimer **69** (Figure 11) with the triazole linkage had a negligible impact on the hybridization properties of modified PNA strands and sequence fidelity during PNA synthesis was maintained. These results indicate the suitability of a triazole *in lieu* of the amide linkage within PNA polymers and its capability with the click-templated coupling of PNA fragments.

Krishna and Caruthers developed the 1,2,3-triazolylphosphonate (TP) internucleotide linkage wherein one of the non-bridging oxygen atoms of the phosphate linkage is replaced by a 1,2,3-triazole moiety, tethered via the C4 of the heterocycle [42]. This was the first time that click-mediated modification at the phosphorus center had been explored, with the exception of a triazole-functionalization at the 5′-phosphate of a DNA strand [138]. The proposed 1,2,3-triazolylphosphonate internucleotide (TP) linkages (**71**, Figure 11) are generated through a two-step process. Initially the ethynylphosphonate internucleotide linkage **70** was introduced at designated sites through conventional phosphoramidite chemistry, followed with the post-synthetic CuAAC to introduce the appropriate azide before deprotection and cleavage of the oligonucleotide from the solid support. By coupling these two steps, chimeric oligonucleotides (16−23 mers) which contain up to six TP modifications as well as other functionalities such as LNA nucleotides, 2′-*O*Me ribonucleotides, and phosphorothioates [139] were synthesized. Nuclease stability assays indicated that these TP-modified ODNs were highly resistant towards exonucleases, whereas melting studies indicated a slight destabilization when a TP-modified ODN was hybridized to its complementary RNA. A fluorescently labeled 16-mer ODN modified with two TP linkages also showed efficient cellular uptake during passive transfection into mammalian cells. This methodology enables the multiple, high-density functionalization of the phosphorus-based backbone of an oligodeoxynucleotide using click chemistry. Various biologically relevant moieties, labeling dyes and diagnostic elements can be introduced at the phosphorus center as opposed to other typically modified areas of DNA nucleosides without compromising the product’s ability to hybridize to its compliment.

### 2.4. Bio-conjugation

Bioconjugation involves conjugating a particular biological scaffold to an existing oligonucleotide that can favorably alter its properties. For example, the poor cell or tissue-specific delivery of synthetic oligonucleotides can be addressed by attaching peptides, lipid derivatives, sugars and other small molecules to natural or modified oligonucleotides [140]. Fluorescent labeling of oligonucleotides enables us to detect, visualize and study the function of the synthetic oligonucleotides and their interactive behavior within biological systems [141]. Many dyes are incorporated post-synthetically, in which a small reactive group is introduced to a DNA or RNA oligonucleotide following deprotection [142]. The CuAAC reaction has been successfully applied in bioconjugation reactions due to its reliability, efficiency, and the stability of alkynes and azides in most physiological and organic conditions.

#### 2.4.1. Nucleic Acids Delivery

**Scheme 14:**
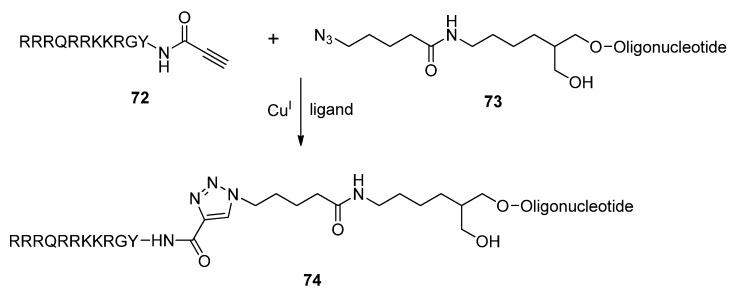
Synthesis of oligonucleotide-Tat peptide conjugates.

In order to enhance the uptake and stability of diagnostic and therapeutic oligonucleotides [143,144], Graham and Brown successfully attached a cell-penetrating peptide (Tat peptide) [145,146] to oligonucleotides via the CuAAC (Scheme 14) [147]. After attaching a click functional group to the target oligonucleotide, the Tat peptide was then functionalized with the opposing click-compatible group. To yield a 3′-azido-modified oligonucleotide (**73**), succinimidyl azidovalerate was synthesized and reacted with an amino-modified solid support. Propiolic acid was coupled to the *N*-terminus of the Tat peptide to form an amide bond which gave the necessary alkyne-modified peptide derivative **72**. The two derivatives were then cyclized to form the resultant conjugated system **74**. 

**Figure 12:**
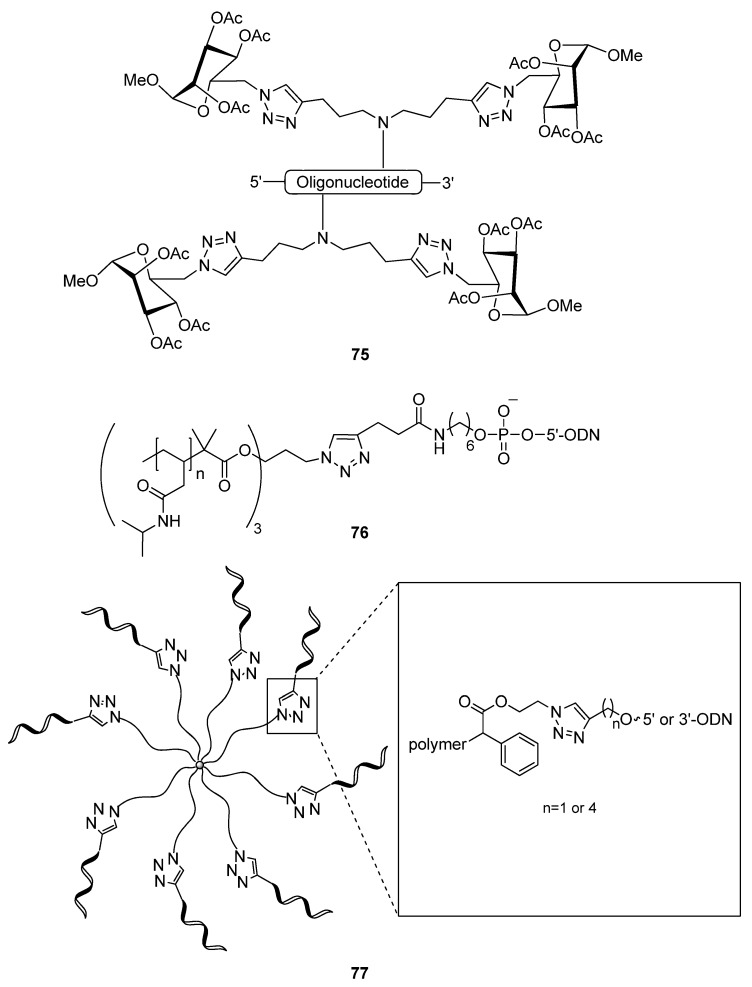
Various structures of triazole-linked oligonucleotide conjugates.

Following the theme of forming peptide- and lipid-based bioconjugate systems, the conjugation of oligonucleotides to carbohydrates is also an effective way to improve the delivery and targeting capability of synthetic oligonucleotides. Kiviniemi and colleagues synthesized 4′-modifed alkyne-derivatized nucleosides which have been utilized in glycoconjuations (**75**, Figure 12) [148]. Azido-derivatized sugar ligands can be efficiently conjugated to the alkynyl groups of these nucleosides via click chemistry. The influence of the 4′-modifications on the melting temperature with DNA and 2′-*O*-methyl RNA targets was studied. The mannose conjugation formed duplexes with complementary 2′-*O*Me RNA, equivalent to the stability of those formed by the corresponding unmodified DNA.

Oligonucleotides are capable of self-aggregating into larger structures due to their sequence-dependent complimentarity and their electronic properties. This next group utilized a technique referred to as atom transfer radical polymerization (ATRP) [149] coupled with click chemistry to create controlled aggregates of DNA co-polymers [150]. They initiated the radical-induced self-assembly of alkyne-functionalized amphiphilic copolymers, followed by their click-conjugation to the 5′-end of alkynylated ssDNA molecules (**76**, Figure 12). Controlled radical polymerization (CRP) techniques such as ATRP can be attractive and facile alternatives to traditional methods of polymerization. Applying these techniques, Averick *et al*. succeeding in coating the core of a star-shaped copolymer comprised of azido groups on its extensions, with 3′- or 5′-alkynyl DNA strands through click-ligation (**77**) [151]. They extended the strategy to form click-conjugated DNA-copolymer hybrids which were concomitantly functionalized with other “clickable” residues containing fluorescent properties. Through these techniques, nucleic acid-based polymers of controlled size and arrangement can be rapidly produced and used for a variety of targeted applications.

#### 2.4.2. Copper-Free Click Ligation

Despite the promising application of CuAAC reaction in oligonucleotide modification, the use of CuAAC in biological systems has been delayed by the fact that copper ions may damage DNA or RNA, thus yielding strand breaks [152]. However, there are several emerging studies that employ uncatalyzed versions of the cycloaddition reaction. The studies mentioned hitherto mainly focus on the use of cyclooctynes, which can undergo strain-promoted azide-alkyne cycloadditions (SPAAC) in the absence of a copper catalyst [153,154].

**Figure 13:**
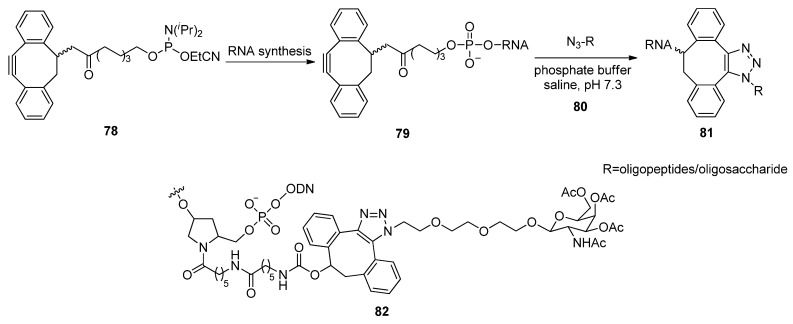
Synthesis and the structure of RNA conjugates via the SPAAC reaction.

The conjugation of a ribonucleotide 16-mer with the cationic amphiphilic peptide penetratin and an anionic hyaluronan tetrasaccharide by means of Cu-free “click” chemistry was reported by the Filippov group [153]. The alkyne-functionalized 16-mer was prepared by automated solid-phase synthesis, using a newly developed strained cyclooctyne phosphoramidite **78** (Figure 13) in the final coupling. The cycloaddition reaction between these alkyne-functionalized RNA oligomers **79** and azide-containing biomolecules **80**, was performed to generate the appropriate synthesized RNA conjugates **81**. The SPAAC reaction was also explored by Manoharan and colleagues who similarly used strained cyclooctynes for Cu-free click cycloadditions to synthesize oligonucleotide-ligand conjugates such as derivative **82** [154]. Both groups demonstrated that an SPAAC reaction could be amenable to forming triazole-linked oligonucleotide-ligand conjugates within biological systems.

#### 2.4.3. Oligonucleotide Labeling

**Figure 14:**
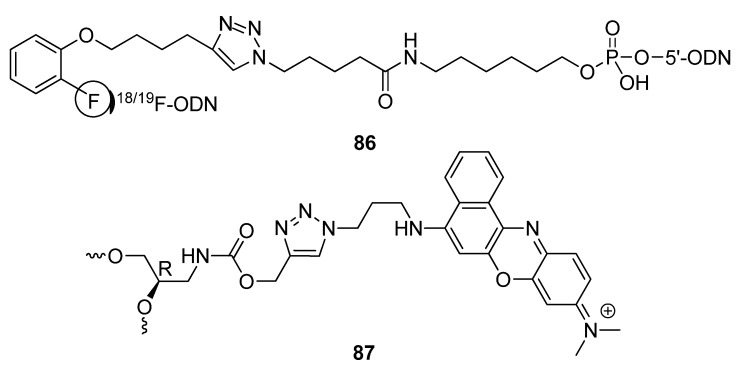
Examples of spin-labels and fluorescent residues linked to oligonucleotides through triazole tethers.

**Scheme 15:**
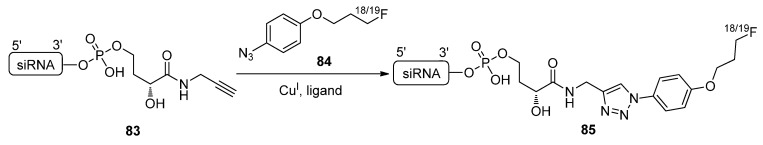
Synthesis of [^18^F]-radiolabeled siRNA by using [^18^F]fluoroazide.

The azide-alkyne click cycloaddition has provided a means to functionalize oligonucleotides with fluorescent properties or to add compounds displaying those properties for clinical diagnosis and to analyze drug-target interactions [155,156]. Inkster and colleagues described the click-mediated attachment of a [^18^F]FPy5yne prosthetic group [157] to a 5′-azide-modified ASO [158]. The ^18^F-labeled oligonucleotide **86** (Figure 14) was obtained with a satisfactory radiochemical yield of 24.6 ± 0.5% (decay corrected), which can then be used for *in vivo* PET imaging studies. Another example of post-synthetically attaching a fluorophore to oligonucleotides for PET imaging studies, the Luxen group are the first to directly label duplex siRNAs through the CuAAC (Scheme 15) [159]. The acetylene-based tether attached to a 3′-end of the duplex **83** served as a scaffold for attaching the azide-functionalized [^18^F]-radiolabelled derivative **84** through click chemistry, producing the conjugate **85**.

For Wagenknecht and colleagues, the CuAAC reaction fit the profile of being the most capable of bioorthogonal reactions required to post-synthetically label a target oligonucleotide with the Nile Blue chromophore [160]. The goal of the group was to study aspects of oligonucleotide structure [161,162] and the effects of chromophore stacking [163] using an acyclic linker scaffold. Incorporating an acyclic 2′-deoxyribonucleoside analogue in the form of a 3-amino-1,2-propanediol linker containing an alkyne tether into synthetic oligonucleotides was the first step towards setting up the click-bioconjugation. The azido-modified Nile Blue chromophore was subsequently click-cyclized with the free acetylenes and tethered through a triazole moiety to the resultant acyclic DNA-mimic scaffold (**87**, Figure 14).

#### 2.4.4. Other Applications of “Click Conjugation”

**Figure 15:**
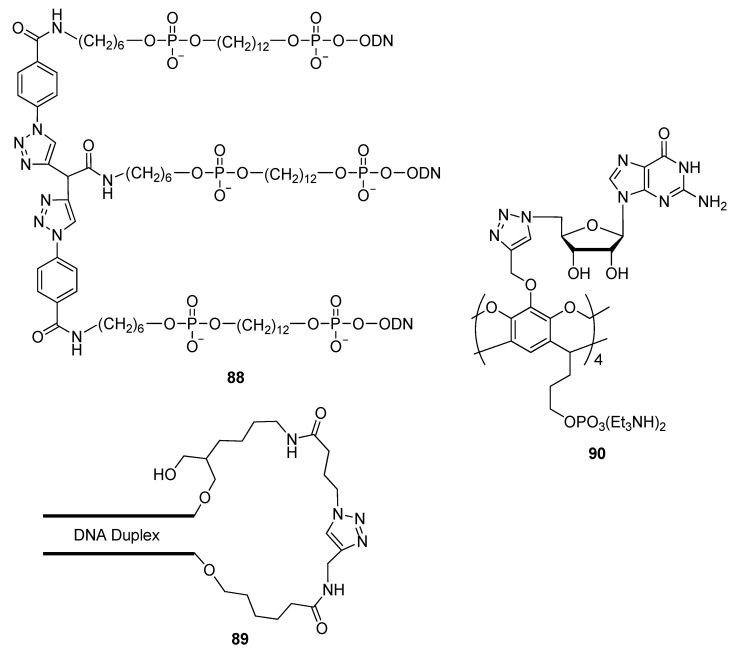
Representatives of other click-conjugated derivatives.

The use of DNA-directed chemistry is becoming increasing popular due its capability of positioning reactants for subsequent reactivity with accuracy and specificity [164]. Since triplex-forming oligonucleotides (TFOs) have been shown to provide an adequate scaffold for controlling specific chemical transformations [165], small molecules employed as TFO binders can be used to influence these TFO-mediated chemical transformations. In an attempt to modulate a di-click conjugation towards the formation of a TFO, a dialkyne-DNA construct and two azido-functionalized DNA strands were covalently paired through a triplex DNA binder [166]. A triplex DNA binder such as naphthylquinoline was utilized by Gothelf and colleagues to successfully form the parallel pyrimidine–purine–pyrimidine triplex **88** (Figure 15). Given the variety of small molecules available as potential DNA binders, the scope of this methodology may be expanded to include other template-directed transformations where conformational control is favorable.

El-Sagheer used click chemistry to ligate two complementary DNA strands to produce an end-sealed DNA duplex **89** (Figure 15) with a triazole-based linkage sealing each end of the duplex [39]. Each single strand had a 5′-terminal alkyne and 3′-azide. The CuAAC reaction was used to link the two strands by covalently joining the 5′-alkyne to the 3′-azide. The resultant constructs were thermally very stable and a fluorescent version remained intact for up to three days in fetal bovine serum. This study illustrates the potential for these triazole end-sealed adducts as decoy protein ligands that can influence gene expression, or find utility in nanotechnology.

In the interest of expanding the repertoire of triazole-modified nanomaterials, the CuAAC reaction was used to synthesize the first water-soluble container-shaped scaffold (cavitand) bearing four inter-locked triazole-linked guanosines (**90**, Figure 15) [167]. Sherman and colleagues used a cavitand-shaped template functionalized with four progargyl ether rim groups and four pendant alkyl phosphate monoesters, serving as precursors for the four-fold click-cyclization with 5′-azido-5′-deoxyguanosine. These compounds may potentially be used as assembly scaffolds for the template-driven construction of G-quadruplexes and other nano-scale structures and devices [168].

**Scheme 16:**
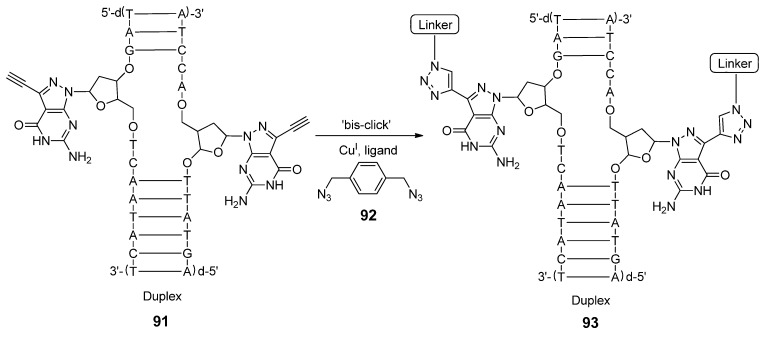
“Bis-click” cross-linking of duplex DNA.

Finally, the Seela group has done extensive work which involves the cross-linking of single-stranded oligonucleotides using click chemistry [38,74,169,170,171]. These studies exemplify the use of alkynyl-modified nucleobases and bis-azides which undergo step-wise click-cyclyzation to form triazole-linked DNA-based adducts. Recently, Seela and colleagues investigated the attributes of the DNA cross-linking process through the CuAAC reaction [38]. They synthesized 7-deaza-alkynylated purine analogues or the corresponding C5-substituted pyrimidine nucleoside analogues, which were specifically incorporated within synthetic duplex oligonucleotides (**91**). Cross-linking was performed using the “bis-click” protocol between an acetylene-modified duplex and the bis-azide linkage **92**, resulting in the chemoselective formation of interstrand cross-linked DNA molecules (**93**, Scheme 16). It was shown that interstrand cross-linked heterodimeric DNA duplexes were stabilized by the resultant cross-linkage.

## 3. Conclusions

It is clearly evident that the field of functionalizing nucleic acids for their application in biological systems or for the development of novel nanomaterials holds a promising future. In the pursuit of finding reactions that can perform simple high-yielding transformations to nucleic acids which deviate from the traditional methods of modification, the Cu(I)-catalyzed version of the Huisgen [3 + 2] cycloaddition satisfies all necessary criteria as an indispensable tool. With the knowledge that azides and alkynes are selectively reactive with each other under a variety of mild or harsh conditions resulting in a stable 1,4-regioisomer of the triazole heterocycle, the CuAAC provides a flexible medium through which nucleic acids can be directly modified. Whether the goal is the creation of novel biopolymers, PCR templates, bioconjugated superstructures, gene-silencing or antiviral agents, the possibilities presented by this modular transformation are endless when considering the number of modifiable sites on natural nucleosides.

## References

[1] Kolb H.C., Finn M.G., Sharpless K.B. (2001). Click chemistry: Diverse chemical function from a few good reactions. Angew. Chem. Int. Ed. Engl..

[2] Amblard F., Cho J.H., Schinazi R.F. (2009). Cu(I)-catalyzed Huisgen azide-alkyne 1,3-dipolar cycloaddition reaction in nucleoside, nucleotide, and oligonucleotide chemistry. Chem. Rev..

[3] Kolb H.C., Sharpless K.B. (2003). The growing impact of click chemistry on drug discovery. Drug Discov. Today.

[4] Meldal M., Tornoe C.W. (2008). Cu-Catalyzed azide-alkyne cycloaddition. Chem. Rev..

[5] Rostovtsev V.V., Green L.G., Fokin V.V., Sharpless K.B. (2002). A stepwise huisgen cycloaddition process: copper(I)-catalyzed regioselective “ligation” of azides and terminal alkynes. Angew. Chem. Int. Ed. Engl..

[6] de Miguel G., Wielopolski M., Schuster D.I., Fazio M.A., Lee O.P., Haley C.K., Ortiz A.L., Echegoyen L., Clark T., Guldi D.M. (2011). Triazole bridges as versatile linkers in electron donor-acceptor conjugates. J. Am. Chem. Soc..

[7] van Dijk M., Nollet M.L., Weijers P., Dechesne A.C., van Nostrum C.F., Hennink W.E., Rijkers D.T.S., Liskamp R.M.J. (2008). Synthesis and characterization of biodegradable peptide-based polymers prepared by microwave-assisted click chemistry. Biomacromolecules.

[8] El-Sagheer A.H., Brown T. (2010). Click chemistry with DNA. Chem. Soc. Rev..

[9] Cho I.S., Kim J., Lim D.H., Ahn H.C., Kim H., Lee K.B., Lee Y.S. (2008). Improved serum stability and biophysical properties of siRNAs following chemical modifications. Biotechnol. Lett..

[10] Miller M.B., Tang Y.W. (2009). Basic concepts of microarrays and potential applications in clinical microbiology. Clin. Microbiol. Rev..

[11] Seo T.S., Bai X.P., Ruparel H., Li Z.M., Turro N.J., Ju J.Y. (2004). Photocleavable fluorescent nucleotides for DNA sequencing on a chip constructed by site-specific coupling chemistry. Proc. Natl. Acad. Sci. USA.

[12] Chevolot Y., Bouillon C., Vidal S., Morvan F., Meyer A., Cloarec J.-P., Jochum A., Praly J.-P., Vasseur J.-J., Souteyrand E. (2007). DNA-Based carbohydrate biochips: A platform for surface glyco-engineering. Angew. Chem. Int. Ed. Engl..

[13] Liu X., Farmerie W., Schuster S., Tan W. (2000). Molecular beacons for DNA biosensors with micrometer to submicrometer dimensions. Anal. Biochem..

[14] Seio K., Takaku Y., Miyazaki K., Kurohagi S., Masaki Y., Ohkubo A., Sekine M. (2009). Synthesis of terminally modified oligonucleotides and their hybridization dependence on the size of the target RNAs. Org. Biomol. Chem..

[15] Izant J.G., Weintraub H. (1984). Inhibition of thymidine kinase gene expression by anti-sense RNA: A molecular approach to genetic analysis. Cell.

[16] Uhlmann E., Peyman A. (1990). Antisense oligonucleotides: A new therapeutic principle. Chem. Rev..

[17] Bumcrot D., Manoharan M., Koteliansky V., Sah D.W.Y. (2006). RNAi therapeutics: A potential new class of pharmaceutical drugs. Nat. Chem. Biol..

[18] Fire A., Xu S.Q., Montgomery M.K., Kostas S.A., Driver S.E., Mello C.C. (1998). Potent and specific genetic interference by double-stranded RNA in *Caenorhabditis elegans*. Nature.

[19] Watts J.K., Deleavey G.F., Damha M.J. (2008). Chemically modified siRNA: Tools and applications. Drug Discov. Today.

[20] Kim S.I., Shin D., Lee H., Ahn B.Y., Yoon Y., Kim M. (2009). Targeted delivery of siRNA against hepatitis C virus by apolipoprotein A-I-bound cationic liposomes. J. Hepatol..

[21] Khaliq S., Khaliq S.A., Zahur M., Ijaz B., Jahan S., Ansar M., Riazuddin S., Hassan S. (2010). RNAi as a new therapeutic strategy against HCV. Biotechnol. Adv..

[22] Elayadi H., Smietana M., Pannecouque C., Leyssen P., Neyts J., Vasseur J.-J., Lazrek H.B. (2010). Straightforward synthesis of triazoloacyclonucleotide phosphonates as potential HCV inhibitors. Bioorg. Med. Chem. Lett..

[23] Corey D.R. (2007). Chemical modification: The key to clinical application of RNA interference?. J. Clin. Invest..

[24] Geary R.S., Henry S.P., Grillone L.R. (2002). Fomivirsen—Clinical pharmacology and potential drug interactions. Clin. Pharmacokinet..

[25] Huffman J.H., Sidwell R.W., Khare G.P., Witkowski J.T., Allen L.B., Robins R.K. (1973). *In-vitro* effect of 1-*β*-D-ribofuranosyl-1,2,4-triazole-3-carboxamide (Virazole, ICN-1229) on deoxyribonucleic acid and ribonucleic acid viruses. Antimicrob. Agents Ch..

[26] El-Sagheer A.H., Brown T. (2010). New strategy for the synthesis of chemically modified RNA constructs exemplified by hairpin and hammerhead ribozymes. Proc. Natl. Acad. Sci. USA.

[27] Mascini M., Palchetti I., Tombelli S. (2012). Nucleic acid and peptide aptamers: Fundamentals and bioanalytical aspects. Angew. Chem. Int. Ed. Engl..

[28] Keefe A.D., Pai S., Ellington A. (2010). Aptamers as therapeutics. Nat. Rev. Drug Discov..

[29] Kaihatsu K., Janowski B.A., Corey D.R. (2004). Recognition of chromosomal DNA by PNAs. Chem. Biol..

[30] Saito Y., Escuret V., Durantel D., Zoulim F., Schinazi R.F., Agrofoglio L.A. (2003). Synthesis of 1,2,3-triazolo-carbanucleoside analogues of ribavirin targeting an HCV in replicon. Bioorg. Med. Chem..

[31] El Akri K., Bougrin K., Balzarini J., Faraj A., Benhida R. (2007). Efficient synthesis and *in vitro* cytostatic activity of 4-substituted triazolyl-nucleosides. Bioorg. Med. Chem. Lett..

[32] Ming X., Seela F. (2012). A nucleobase-discriminating pyrrolo-dC click adduct designed for DNA fluorescence mismatch sensing. Chem. Eur. J..

[33] Xiong Z., Qiu X.-L., Huang Y., Qing F.-L. (2011). Regioselective synthesis of 5-trifluoromethyl-1,2,3-triazole nucleoside analogues via TBS-directed 1,3-dipolar cycloaddition reaction. J. Fluorine Chem..

[34] Gramlich P.M.E., Warncke S., Gierlich J., Carell T. (2008). Click-click-click: Single to triple modification of DNA. Angew. Chem. Int. Ed. Engl..

[35] Ostergaard M.E., Guenther D.C., Kumar P., Baral B., Deobald L., Paszczynski A.J., Sharma P.K., Hrdlicka P.J. (2010). Pyrene-functionalized triazole-linked 2′-deoxyuridines—probes for discrimination of single nucleotide polymorphisms (SNPs). Chem. Commun..

[36] Dodd D.W., Swanick K.N., Price J.T., Brazeau A.L., Ferguson M.J., Jones N.D., Hudson R.H.E. (2010). Blue fluorescent deoxycytidine analogues: Convergent synthesis, solid-state and electronic structure, and solvatochromism. Org. Biomol. Chem..

[37] Seela F., Ingale S.A. (2010). Double click” reaction on 7-deazaguanine DNA: Synthesis and excimer fluorescence of nucleosides and oligonucleotides with branched side chains decorated with proximal pyrenes. J. Org. Chem..

[38] Xiong H., Seela F. (2012). Cross-linked DNA: Site-selective “click” ligation in duplexes with bis-azides and stability changes caused by internal cross-links. Bioconjug. Chem..

[39] El-Sagheer A.H. (2009). Very stable end-sealed double stranded DNA by click chemistry. Nucleos. Nucleot. Nucl..

[40] Jacobsen M.F., Ravnsbaek J.B., Gothelf K.V. (2010). Small molecule induced control in duplex and triplex DNA-directed chemical reactions. Org. Biomol. Chem..

[41] Seela F., Xiong H., Budow S. (2010). Synthesis and ‘double click’ density functionalization of 8-aza-7-deazaguanine DNA bearing branched side chains with terminal triple bonds. Tetrahedron.

[42] Krishna H., Caruthers M.H. (2012). Alkynyl Phosphonate DNA: A versatile “click” able backbone for DNA-based biological applications. J. Am. Chem. Soc..

[43] Peacock H., Maydanovych O., Beal P.A. (2010). *N*^2^-Modified 2-aminopurine ribonucleosides as minor-groove-modulating adenosine replacements in duplex RNA. Org. Lett..

[44] Ohtsuka E., Ikehara M., Soll D. (1982). Recent developments in the chemical synthesis of polynucleotides. Nucleic Acids Res..

[45] Caruthers M.H., Barone A.D., Beaucage S.L., Dodds D.R., Fisher E.F., McBride L.J., Matteucci M., Stabinsky Z., Tang J.Y. (1987). Chemical synthesis of deoxyoligonucleotides by the phosphoramidite method. Method. Enzymol..

[46] Wilson J.N., Kool E.T. (2006). Fluorescent DNA base replacements: reporters and sensors for biological systems. Org. Biomol. Chem..

[47] Peacock H., Fostvedt E., Beal P.A. (2010). Minor-groove-modulating adenosine replacements control protein binding and RNAi activity in siRNAs. ACS Chem. Biol..

[48] Jorgensen A.S., Shaikh K.I., Enderlin G., Ivarsen E., Kumar S., Nielsen P. (2011). The synthesis of double-headed nucleosides by the CuAAC reaction and their effect in secondary nucleic acid structures. Org. Biomol. Chem..

[49] Okamoto A., Saito Y., Saito I. (2005). Design of base-discriminating fluorescent nucleosides. J. Photoch. Photobio. C.

[50] Addepalli H., Meena, Peng C.G., Wang G., Fan Y., Charisse K., Jayaprakash K.N., Rajeev K.G., Pandey R.K., Lavine G., Zhang L. (2010). Modulation of thermal stability can enhance the potency of siRNA. Nucleic Acids Res..

[51] Chittepu P., Sirivolu V.R., Seela F. (2008). Nucleosides and oligonucleotides containing 1,2,3-triazole residues with nucleobase tethers: Synthesis via the azide-alkyne ‘click’ reaction. Bioorg. Med. Chem..

[52] Ding P., Wunnicke D., Steinhoff H.-J., Seela F. (2010). Site-directed spin-labeling of DNA by the azide-alkyne ‘click’ reaction: Nanometer distance measurements on 7-deaza-2′-deoxyadenosine and 2′-deoxyuridine nitroxide conjugates spatially separated or linked to a ‘dA-dT’ base pair. Chem. Eur. J..

[53] Beyer C., Wagenknecht H.-A. (2010). *In situ* azide formation and “click” reaction of nile red with DNA as an alternative postsynthetic route. Chem. Commun..

[54] Park S.M., Yang H., Park S.-K., Kim H.M., Kim B.H. (2010). Design, synthesis, and anticancer activities of novel perfluoroalkyltriazole-appended 2′-deoxyuridines. Bioorg. Med. Chem. Lett..

[55] Montagu A., Roy V., Balzarini J., Snoeck R., Andrei G., Agrofoglio L.A. (2011). Synthesis of new C5-(1-substituted-1,2,3-triazol-4 or 5-yl)-2′-deoxyuridines and their antiviral evaluation. Eur. J. Med. Chem..

[56] Streeter D.G., Witkowski J.T., Khare G.P., Sidwell R.W., Bauer R.J., Robins R.K., Simon L.N. (1973). Mechanism of action of 1-*β*-D-ribofuranosyl-1,2,4-triazole-3-carboxamide (Virazole), a new broad-spectrum antiviral agent. Proc. Natl. Acad. Sci. USA.

[57] Lin J., Roy V., Wang L., You L., Agrofoglio L.A., Deville-Bonne D., McBrayer T.R., Coats S.J., Schinazi R.F., Eriksson S. (2010). 3′-(1,2,3-Triazol-1-yl)-3′-deoxythymidine analogs as substrates for human and *Ureaplasma parvum* thymidine kinase for structure-activity investigations. Bioorg. Med. Chem..

[58] Yu B., Zhao X., Lee L.J., Lee R.J. (2009). Targeted delivery systems for oligonucleotide therapeutics. AAPS J..

[59] Crooke S.T. (1998). Basic Principles of Antisense Therapeutics.

[60] Stephenson M.L., Zamecnik P.C. (1978). Inhibition of Rous sarcoma viral RNA translation by a specific oligodeoxyribonucleotide. Proc. Natl. Acad. Sci. USA.

[61] Deleavey G.F., Damha M.J. (2012). Designing chemically modified oligonucleotides for targeted gene silencing. Chem. Biol..

[62] Andersen N.K., Dossing H., Jensen F., Vester B., Nielsen P. (2011). Duplex and triplex formation of mixed pyrimidine oligonucleotides with stacking of phenyl-triazole moieties in the major groove. J. Org. Chem..

[63] Wagner R.W., Matteucci M.D., Lewis J.G., Gutierrez A.J., Moulds C., Froehler B.C. (1993). Antisense gene inhibition by oligonucleotides containing C-5 propyne pyrimidines. Science.

[64] Gutierrez A.J., Matteucci M.D., Grant D., Matsumura S., Wagner R.W., Froehler B.C. (1997). Antisense gene inhibition by C-5-substituted deoxyuridine-containing oligodeoxynucleotides. Biochemistry.

[65] Kumar P., Chandak N., Nielsen P., Sharma P.K. (2012). Sulfonamide bearing oligonucleotides: Simple synthesis and efficient RNA recognition. Bioorg. Med. Chem..

[66] Kocalka P., Andersen N.K., Jensen F., Nielsen P. (2007). Synthesis of 5-(1,2,3-triazol-4-yl)-2′-deoxyuridines by a click chemistry approach: Stacking of triazoles in the major groove gives increased nucleic acid duplex stability. ChemBioChem.

[67] Whiting M., Muldoon J., Lin Y.C., Silverman S.M., Lindstrom W., Olson A.J., Kolb H.C., Finn M.G., Sharpless K.B., Elder J.H. (2006). Inhibitors of HIV-1 protease by using in situ click chemistry. Angew. Chem. Int. Ed. Engl..

[68] Robins M.J., Barr P.J. (1983). Nucleic acid related compounds. 39. Efficient conversion of 5-iodo to 5-alkynyl and derived 5-substituted uracil bases and nucleosides. J. Org. Chem..

[69] Bramsen J.B., Laursen M.B., Nielsen A.F., Hansen T.B., Bus C., Langkjaer N., Babu B.R., Hojland T., Abramov M., Van Aerschot A. (2009). A large-scale chemical modification screen identifies design rules to generate siRNAs with high activity, high stability and low toxicity. Nucleic Acids Res..

[70] Robbins M., Judge A., MacLachlan I. (2009). siRNA and innate immunity. Oligonucleotides.

[71] Chang K.Y., Ramos A. (2005). The double-stranded RNA-binding motif, a versatile macromolecular docking platform. FEBS J..

[72] Tian B., Bevilacqua P.C., Diegelman-Parente A., Mathews M.B. (2004). The double-stranded-RNA-binding motif: Interference and much more. Nat. Rev. Mol. Cell Biol..

[73] Qing G., Xiong H., Seela F., Sun T. (2010). Spatially controlled DNA nanopatterns by “click” chemistry using oligonucleotides with different anchoring sites. J. Am. Chem. Soc..

[74] Seela F., Xiong H., Leonard P., Budow S. (2009). 8-Aza-7-deazaguanine nucleosides and oligonucleotides with octadiynyl side chains: Synthesis, functionalization by the azide-alkyne ‘click’ reaction and nucleobase specific fluorescence quenching of coumarin dye conjugates. Org. Biomol. Chem..

[75] Manalo M.N., Perez L.M., LiWang A. (2007). Hydrogen-bonding and π-π base-stacking interactions are coupled in DNA, as suggested by calculated and experimental *trans* H-bond deuterium isotope shifts. J. Am. Chem. Soc..

[76] Kellner S., Seidu-Larry S., Burhenne J., Motorin Y., Helm M. (2011). A multifunctional bioconjugate module for versatile photoaffinity labeling and click chemistry of RNA. Nucleic Acids Res..

[77] Onizuka K., Shibata A., Taniguchi Y., Sasaki S. (2011). Pin-point chemical modification of RNA with diverse molecules through the functionality transfer reaction and the copper-catalyzed azide-alkyne cycloaddition reaction. Chem. Commun..

[78] Schiemann O., Prisner T.F. (2007). Long-range distance determinations in biomacromolecules by EPR spectroscopy. Q Rev. Biophys..

[79] Bushmakina N.G., Misharin A.Y. (1986). A simple synthesis of 4-amino-2,2,6,6-tetramethyl-1-piperidinyloxy radical. Synthesis-Stuttgart.

[80] Ametamey S.M., Honer M., Schubiger P.A. (2008). Molecular imaging with PET. Chem. Rev..

[81] Miller P.W., Long N.J., Vilar R., Gee A.D. (2008). Synthesis of ^11^C, ^18^F, ^15^O, and ^13^N Radiolabels for Positron Emission Tomograpy. Angew. Chem. Int. Ed. Engl..

[82] Kuboyama T., Nakahara M., Yoshino M., Cui Y., Sako T., Wada Y., Imanishi T., Obika S., Watanabe Y., Suzuki M. (2011). Stoichiometry-focused ^18^F-labeling of alkyne-substituted oligodeoxynucleotides using azido([^18^F]fluoromethyl)benzenes by Cu-catalyzed Huisgen reaction. Bioorg. Med. Chem..

[83] Inoue H., Imura A., Ohtsuka E. (1987). Synthesis of dodecadeoxyribonucleotides containing a pyrrolo[2,3-d]pyrimidine nucleoside and their base-pairing ability. Nippon Kagaku Kaishi.

[84] Dierckx A., Diner P., El-Sagheer A.H., Kumar J.D., Brown T., Grotli M., Wilhelmsson L.M. (2011). Characterization of photophysical and base-mimicking properties of a novel fluorescent adenine analogue in DNA. Nucleic Acids Res..

[85] Dyrager C., Borjesson K., Diner P., Elf A., Albinsson B., Wilhelmsson L.M., Grotli M. (2009). Synthesis and photophysical characterisation of fluorescent 8-(1*H*-1,2,3-triazol-4-yl)adenosine derivatives. Eur. J. Org. Chem..

[86] Seela F., Peng X., Budow S. (2007). Advances in the synthesis of 7-deazapurine-pyrrolo[2,3-d]pyrimidine-2 ′-deoxyribonucleosides including D- and L-enantiomers, fluoro derivatives and 2′,3′-dideoxyribonucleosides. Curr. Org. Chem..

[87] Winnik F.M. (1993). Photophysics of preassociated pyrenes in aqueous polymer-solutions and in other organized media. Chem. Rev..

[88] Ming X., Ding P., Leonard P., Budow S., Seela F. (2012). Parallel-stranded DNA: Enhancing duplex stability by the ‘G-clamp ’ and a pyrrolo-dC derivative. Org. Biomol. Chem..

[89] Lin K.Y., Matteucci M.D. (1998). A cytosine analogue capable of clamp-like binding to a guanine in helical nucleic acids. J. Am. Chem. Soc..

[90] Koszytkowska-Stawinska M., Mironiuk-Puchalska E., Rowicki T. (2012). Synthesis of 1,2,3-triazolo-nucleosides via the post-triazole N-alkylation. Tetrahedron.

[91] De Clercq E., Holy A. (2005). Acyclic nucleoside phosphonates: A key class of antiviral drugs. Nat. Rev. Drug Discov..

[92] Ganesan M., Muraleedharan K.M. (2010). Synthesis of *β*-hydroxyphosphonate and 1,2-dihydroxy acyclic nucleoside analogs via 1,3-dipolar cycloaddition strategy. Nucleos. Nucleot. Nucl..

[93] Trakossas S., Coutouli-Argyropoulou E., Hadjipavlou-Litina D.J. (2011). Synthesis of modified triazole nucleosides possessing one or two base moieties via a click chemistry approach. Tetrahedron Lett..

[94] Duschinsky R., Pleven E., Heidelberger C. (1957). The synthesis of 5-fluoropyrimidines. J. Am. Chem. Soc..

[95] Lakshman M.K., Singh M.K., Parrish D., Balachandran R., Day B.W. (2010). Azide-tetrazole equilibrium of C-6 azidopurine nucleosides and their ligation reactions with alkynes. J. Org. Chem..

[96] Mathew S.C., By Y., Berthault A., Virolleaud M.-A., Carrega L., Chouraqui G., Commeiras L., Condo J., Attolini M., Gaudel-Siri A. (2010). Expeditious synthesis and biological evaluation of new C-6 1,2,3-triazole adenosine derivatives A1 receptor antagonists or agonists. Org. Biomol. Chem..

[97] Driowya M., Puissant A., Robert G., Auberger P., Benhida R., Bougrin K. (2012). Ultrasound-assisted one-pot synthesis of anti-CML nucleosides featuring 1,2,3-triazole nucleobase under iron-copper catalysis. Ultrason. Sonochem..

[98] Kolganova N.A., Florentiev V.L., Chudinov A.V., Zasedatelev A.S., Timofeev E.N. (2011). Simple and stereoselective preparation of an 4-(aminomethyl)-1,2,3-triazolyl nucleoside phosphoramidite. Chem. Biodivers..

[99] Hou S., Liu W., Ji D., Zhao Z. (2011). Efficient synthesis of triazole moiety-containing nucleotide analogs and their inhibitory effects on a malic enzyme. Bioorg. Med. Chem. Lett..

[100] Bhatt B., Thomson R.J., von Itzstein M. (2011). An efficient approach to 2,5-anhydro-glucitol-based 1′-homo-*N*-nucleoside mimetics. Tetrahedron Lett..

[101] Alvarez R., Velazquez S., Sanfelix A., Aquaro S., Declercq E., Perno C.F., Karlsson A., Balzarini J., Camarasa M.J. (1994). 1,2,3-Triazole-[2′,5′-bis-*O*-(tert-butyldimethylsilyl)-beta-D-ribofuranosyl]-3′-spiro-5′′-(4′′-amino-1′′,2′′-oxathiol 2′′,2′′-dioxide) (TSAO) analogs: Synthesis and anti-HIV-1 activity. J. Med. Chem..

[102] Singh S.K., Nielsen P., Koshkin A.A., Wengel J. (1998). LNA (locked nucleic acids): Synthesis and high-affinity nucleic acid recognition. Chem. Commun..

[103] Van Poecke S., Negri A., Gago F., Van Daele I., Solaroli N., Karlsson A., Balzarini J., Van Calenbergh S. (2010). 3′-[4-Aryl-(1,2,3-triazol-1-yl)]-3′-deoxythymidine Analogues as Potent and Selective Inhibitors of Human Mitochondrial Thymidine Kinase. J. Med. Chem..

[104] Yu J.-L., Wu Q.-P., Zhang Q.-S., Xi X.-D., Liu N.-N., Li Y.-Z., Liu Y.-H., Yin H.-Q. (2010). Synthesis and antitumor activity of novel 2′,3′-diethanethio-2′,3′,5′-trideoxy-5′-triazolonucleoside analogues. Eur. J. Med. Chem..

[105] Baraniak D., Kacprzak K., Celewicz L. (2011). Synthesis of 3′-azido-3′-deoxythymidine (AZT)–*Cinchona* alkaloid conjugates via click chemistry: Toward novel fluorescent markers and cytostatic agents. Bioorg. Med. Chem. Lett..

[106] Yamada T., Peng C.G., Matsuda S., Addepalli H., Jayaprakash K.N., Alam M.R., Mills K., Maier M.A., Charisse K., Sekine M. (2011). Versatile site-specific conjugation of small molecules to siRNA using click chemistry. J. Org. Chem..

[107] Roy V., Obikhod A., Zhang H.-W., Coats S.J., Herman B.D., Sluis-Cremer N., Agrofoglio L.A., Schinazi R.F. (2011). Synthesis and anti-HIV evaluation of 3′-triazolo nucleosides. Nucleos. Nucleot. Nucl..

[108] Fauster K., Hartl M., Santner T., Aigner M., Kreutz C., Bister K., Ennifar E., Micura R. (2012). 2′-Azido RNA, a versatile tool for chemical biology: Synthesis, X-ray structure, siRNA applications, click labeling. ACS Chem. Biol..

[109] Kaczmarek O., Scheidt H.A., Bunge A., Foese D., Karsten S., Arbuzova A., Huster D., Liebscher J. (2010). 2′-Linking of lipids and other functions to uridine through 1,2,3-triazoles and membrane anchoring of the amphiphilic products. Eur. J. Org. Chem..

[110] Sau S.P., Hrdlicka P.J. (2012). C2′-Pyrene-functionalized triazole-linked DNA: Universal DNA/RNA hybridization probes. J. Org. Chem..

[111] Johansson K., Ramaswamy S., Ljungcrantz C., Knecht W., Piskur J., Munch-Petersen B., Eriksson S., Eklund H. (2001). Structural basis for substrate specificities of cellular deoxyribonucleoside kinases. Nat. Struct. Biol..

[112] Hallek M., Wanders L., Strohmeyer S., Emmerich B. (1992). Thymidine kinase: A tumor-marker with prognostic value for non-Hodgkin’s lymphoma and a broad range of potential clinical applications. Ann. Hematol..

[113] Eriksson S., Munch-Petersen B., Johansson K., Eklund H. (2002). Structure and function of cellular deoxyribonucleoside kinases. Cell. Mol. Life Sci..

[114] Christensen M.S., Madsen C.M., Nielsen P. (2007). Synthesis and modelling of DNA junction and minor groove zipper motifs incorporating the double-headed nucleoside 5′(*S*)-*C*-(thymine-1-ylmethyl)thymidine. Org. Biomol. Chem..

[115] Aigner M., Hartl M., Fauster K., Steger J., Bister K., Micura R. (2011). Chemical synthesis of site-specifically 2′-azido-modified RNA and potential applications for bioconjugation and RNA interference. ChemBioChem.

[116] Staudinger H., Meyer J. (1919). On new organic phosphorus bonding III phosphine methylene derivatives and phosphinimine. Helv. Chim. Acta.

[117] Micura R. (1999). Cyclic Oligoribonucleotides (RNA) by solid-phase synthesis. Chem. Eur. J..

[118] Birks J.B. (1975). Excimers. Rep. Prog. Phys..

[119] Caruthers M.H. (1991). Chemical synthesis of DNA and DNA analogs. Accounts Chem. Res..

[120] Paredes E., Das S.R. (2011). Click chemistry for rapid labeling and ligation of RNA. ChemBioChem.

[121] Chandrasekhar S., Srihari P., Nagesh C., Kiranmai N., Nagesh N., Idris M.M. (2010). Synthesis of readily accessible triazole-linked dimer deoxynucleoside phosphoramidite for solid-phase oligonucleotide synthesis. Synthesis-Stuttgart.

[122] Chandrasekhar S., Rambabu C., Reddy A.S. (2008). Spirastrellolide B: the synthesis of southern (C_9_–C_25_) region. Org. Lett..

[123] Corey E.J., Fuchs P.L. (1972). Synthetic method for formyl->ethynyl conversion (RCHO->RC=CH or RC=CR′). Tetrahedron Lett..

[124] Varizhuk A., Chizhov A., Smirnov I., Kaluzhny D., Florentiev V. (2012). Triazole-Linked Oligonucleotides with Mixed-Base Sequences: Synthesis and Hybridization Properties. Eur. J. Org. Chem..

[125] El-Sagheer A.H., Brown T. (2009). Synthesis and Polymerase Chain Reaction Amplification of DNA Strands Containing an Unnatural Triazole Linkage. J. Am. Chem. Soc..

[126] Montevecchi P.C., Manetto A., Navacchia M.L., Chatgilialoglu C. (2004). Thermal decomposition of the *tert*-butyl perester of thymidine-5′-carboxylic acid. Formation and fate of the pseudo-C4′ radical. Tetrahedron.

[127] Zhang J.C., Matteucci M.D. (1999). Synthesis of a *N*-acylsulfamide linked dinucleoside and its incorporation into an oligonucleotide. Bioorg. Med. Chem. Lett..

[128] El-Sagheer A.H., Sanzone A.P., Gao R., Tavassoli A., Brown T. (2011). Biocompatible artificial DNA linker that is read through by DNA polymerases and is functional in *Escherichia coli*. Proc. Natl. Acad. Sci. USA.

[129] Dallmann A., El-Sagheer A.H., Dehmel L., Mugge C., Griesinger C., Ernsting N.P., Brown T. (2011). Structure and Dynamics of Triazole-Linked DNA: Biocompatibility Explained. Chem. Eur. J..

[130] Mutisya D., Selvam C., Kennedy S.D., Rozners E. (2011). Synthesis and properties of triazole-linked RNA. Bioorg. Med. Chem. Lett..

[131] Isobe H., Fujino T., Yamazaki N., Guillot-Nieckowski M., Nakamura E. (2008). Triazole-linked analogue of deoxyribonucleic acid (^TL^DNA): Design, synthesis, and double-strand formation with natural DNA. Org. Lett..

[132] Fujino T., Endo K., Yamazaki N., Isobe H. (2012). Synthesis of triazole-linked analogues of RNA (^TL^RNA). Chem. Lett..

[133] Efthymiou T.C., Desaulniers J.-P. (2011). Synthesis and properties of oligonucleotides that contain a triazole-linked nucleic acid dimer. J. Heterocycl. Chem..

[134] Efthymiou T.C., Vanthi H., Oentoro J., Peel B., Desaulniers J.-P. (2012). Efficient synthesis and cell-based silencing activity of siRNAs that contain triazole backbone linkages. Bioorg. Med. Chem. Lett..

[135] Nielsen P.E., Egholm M., Berg R.H., Buchardt O. (1991). Sequence-selective recognition of DNA by strand displacement with a thymine-substituted polyamide. Science.

[136] Chouikhi D., Barluenga S., Winssinger N. (2010). Clickable peptide nucleic acids (cPNA) with tunable affinity. Chem. Commun..

[137] Thomson S.A., Josey J.A., Cadilla R., Gaul M.D., Hassman C.F., Luzzio M.J., Pipe A.J., Reed K.L., Ricca D.J., Wiethe R.W. (1995). Fmoc mediated synthesis of peptide nucleic acids. Tetrahedron.

[138] Kumar R., El-Sagheer A., Tumpane J., Lincoln P., Wilhelmsson L.M., Brown T. (2007). Template-directed oligonucleotide strand ligation, covalent intramolecular DNA circularization and catenation using click chemistry. J. Am. Chem. Soc..

[139] Eckstein F. (1966). Nucleoside phosphorothioates. J. Am. Chem. Soc..

[140] Singh Y., Spinelli N., Defrancq E. (2008). Chemical strategies for oligonucleotide-conjugates synthesis. Curr. Org. Chem..

[141] Sameiro M., Goncalves T. (2009). Fluorescent labeling of biomolecules with organic probes. Chem. Rev..

[142] Best M.D. (2009). Click chemistry and bioorthogonal reactions: Unprecedented selectivity in the labeling of biological molecules. Biochemistry.

[143] Moulton H.M., Nelson M.H., Hatlevig S.A., Reddy M.T., Iversen P.L. (2004). Cellular uptake of antisense morpholino oligomers conjugated to arginine-rich peptides. Bioconjug. Chem..

[144] Englisch U., Gauss D.H. (1991). Chemically modified oligonucleotides as probes and inhibitors. Angew. Chem. Int. Ed. Engl..

[145] Nitin N., Santangelo P.J., Kim G., Nie S.M., Bao G. (2004). Peptide-linked molecular beacons for efficient delivery and rapid mRNA detection in living cells. Nucleic Acids Res..

[146] Eguchi A., Akuta T., Okuyama H., Senda T., Yokoi H., Inokuchi H., Fujita S., Hayakawa T., Takeda K., Hasegawa M. (2001). Protein transduction domain of HIV-1 tat protein promotes efficient delivery of DNA into mammalian cells. J. Biol. Chem..

[147] Brown S.D., Graham D. (2010). Conjugation of an oligonucleotide to Tat, a cell-penetrating peptide, via click chemistry. Tetrahedron Lett..

[148] Kiviniemi A., Virta P., Lonnberg H. (2010). Solid-supported synthesis and click conjugation of 4′-*C*-alkyne functionalized oligodeoxyribonucleotides. Bioconjug. Chem..

[149] Matyjaszewski K. (2012). Atom transfer radical polymerization: From mechanisms to applications. Isr. J. Chem..

[150] Pan P.J., Fujita M., Ooi W.Y., Sudesh K., Takarada T., Goto A., Maeda M. (2011). DNA-functionalized thermoresponsive bioconjugates synthesized via ATRP and click chemistry. Polymer.

[151] Averick S., Paredes E., Li W.W., Matyjaszewski K., Das S.R. (2011). Direct DNA conjugation to star polymers for controlled reversible assemblies. Bioconjug. Chem..

[152] Jewett J.C., Bertozzi C.R. (2010). Cu-free click cycloaddition reactions in chemical biology. Chem. Soc. Rev..

[153] van Delft P., Meeuwenoord N.J., Hoogendoorn S., Dinkelaar J., Overkleeft H.S., van der Marel G.A., Filippov D.V. (2010). Synthesis of oligoribonucleic acid conjugates using a cyclooctyne phosphoramidite. Org. Lett..

[154] Jayaprakash K.N., Peng C.G., Butler D., Varghese J.P., Maier M.A., Rajeev K.G., Manoharan M. (2010). Non-nucleoside building blocks for copper-assisted and copper-free click chemistry for the efficient synthesis of RNA conjugates. Org. Lett..

[155] Boisgard R., Kuhnast B., Vonhoff S., Younes C., Hinnen F., Verbavatz J.M., Rousseau B., Furste J., Wlotzka B., Dolle F. (2005). *In vivo* biodistribution and pharmacokinetics of ^18^F-labelled Spiegelmers: A new class of oligonucleotidic radiopharmaceuticals. Eur. J. Nucl. Med. Mol. I..

[156] Viel T., Boisgard R., Kuhnast B., Jego B., Siquier-Pernet K., Hinnen F., Dolle F., Tavitian B. (2008). Molecular imaging study on *in vivo* distribution and pharmacokinetics of modified small interfering RNAs (siRNAs). Oligonucleotides.

[157] Inkster J.A.H., Guerin B., Ruth T.J., Adam M.J. (2008). Radiosynthesis and bioconjugation of [^18^F]FPy5yne, a prosthetic group for the ^18^F labeling of bioactive peptides. J. Labelled Compd. Rad..

[158] Inkster J.A.H., Adam M.J., Storr T., Ruth T.J. (2009). Labeling of an antisense oligonucleotide with [^18^F]FPy5yne. Nucleos. Nucleot. Nucl..

[159] Mercier F., Paris J., Kaisin G., Thonon D., Flagothier J., Teller N., Lemaire C., Luxen A. (2011). General method for labeling siRNA by click chemistry with fluorine-18 for the purpose of PET imaging. Bioconjug. Chem..

[160] Lachmann D., Berndl S., Wolfbeis O.S., Wagenknecht H.-A. (2010). Synthetic incorporation of Nile Blue into DNA using 2′-deoxyriboside substitutes: Representative comparison of (*R*)- and (*S*)-aminopropanediol as an acyclic linker. Beilstein J. Org. Chem..

[161] Takarada T., Tamaru D., Liang X.G., Asanuma H., Komiyama M. (2001). L-Threoninol as a chiral linker of azobenzene for the effective photo-regulation of DNA triplex formation. Chem. Lett..

[162] Asanuma H., Takarada T., Yoshida T., Tamaru D., Liang X.G., Komiyama M. (2001). Enantioselective incorporation of azobenzenes into oligodeoxyribonucleotide for effective photoregulation of duplex formation. Angew. Chem. Int. Ed. Engl..

[163] Shi Y., Machida K., Kuzuya A., Komiyama M. (2005). Design of phosphoramidite monomer for optimal incorporation of functional intercalator to main chain of oligonucleotide. Bioconjug. Chem..

[164] Li X.Y., Liu D.R. (2004). DNA-templated organic synthesis: Nature ’s strategy for controlling chemical reactivity applied to synthetic molecules. Angew. Chem. Int. Ed. Engl..

[165] Chen Y., Mao C. (2004). Reprogramming DNA-directed reactions on the basis of a DNA conformational change. J. Am. Chem. Soc..

[166] Jacobsen M.F., Ravnsbaek J.B., Gothelf K.V. (2010). Small molecule induced control in duplex and triplex DNA-directed chemical reactions. Org. Biomol. Chem..

[167] Nikan M., Bare G.A.L., Sherman J.C. (2011). Synthesis of a water-soluble triazole-linked cavitand-guanosine conjugate. Tetrahedron Lett..

[168] Ou T.-M., Lu Y.-J., Tan J.-H., Huang Z.-S., Wong K.-Y., Gu L.-Q. (2008). G-quadruplexes: Targets in anticancer drug design. ChemMedChem.

[169] Seela F., Sirivolu V.R. (2006). DNA containing side chains with terminal triple bonds: Base-pair stability and functionalization of alkynylated pyrimidines and 7-deazapurines. Chem. Biodivers..

[170] Seela F., Sirivolu V.R. (2007). Nucleosides and oligonucleotides with diynyl side chains: Base pairing and functionalization of 2′-deoxyuridine derivatives by the copper(I)-catalyzed alkyne-azide ′click′ cycloaddition. Helv. Chim. Acta.

[171] Seela F., Sirivolu V.R., Chittepu P. (2008). Modification of DNA with octadiynyl side chains: Synthesis, base pairing, and formation of fluorescent coumarin dye conjugates of four nucleobases by the alkyne–azide “click” reaction. Bioconjug. Chem..

